# T6SS secretes an LPS-binding effector to recruit OMVs for exploitative competition and horizontal gene transfer

**DOI:** 10.1038/s41396-021-01093-8

**Published:** 2021-08-25

**Authors:** Changfu Li, Lingfang Zhu, Dandan Wang, Zhiyan Wei, Xinwei Hao, Zhuo Wang, Tengfei Li, Lei Zhang, Zhiqiang Lu, Mingxiu Long, Yao Wang, Gehong Wei, Xihui Shen

**Affiliations:** 1 State Key Laboratory of Crop Stress Biology for Arid Areas, Shaanxi Key Laboratory of Agricultural and Environmental Microbiology, College of Life Sciences, Northwest A&F University, Yangling, Shaanxi China; 2 Department of Entomology, College of Plant Protection, Northwest A&F University, Yangling, Shaanxi China; 3 Department of Biochemistry and Molecular Biology, College of Life Sciences, Northwest A&F University, Yangling, Shaanxi China; 4 College of Grassland and Agriculture, Northwest A&F University, Yangling, Shaanxi China

**Keywords:** Microbial communities, Microbial ecology, Metals

## Abstract

Outer membrane vesicles (OMVs) can function as nanoscale vectors that mediate bacterial interactions in microbial communities. How bacteria recognize and recruit OMVs inter-specifically remains largely unknown, thus limiting our understanding of the complex physiological and ecological roles of OMVs. Here, we report a ligand-receptor interaction-based OMV recruitment mechanism, consisting of a type VI secretion system (T6SS)-secreted lipopolysaccharide (LPS)-binding effector TeoL and the outer membrane receptors CubA and CstR. We demonstrated that *Cupriavidus necator* T6SS1 secretes TeoL to preferentially associate with OMVs in the extracellular milieu through interactions with LPS, one of the most abundant components of OMVs. TeoL associated with OMVs can further bind outer membrane receptors CubA and CstR, which tethers OMVs to the recipient cells and allows cargo to be delivered. The LPS-mediated mechanism enables bacterial cells to recruit OMVs derived from different species, and confers advantages to bacterial cells in iron acquisition, interbacterial competition, and horizontal gene transfer (HGT). Moreover, our findings provide multiple new perspectives on T6SS functionality in the context of bacterial competition and HGT, through the recruitment of OMVs.

## Introduction

Outer membrane vesicles (OMVs) are nanospherical proteoliposomes (20–400 nm diameter) continually released from the outer membrane of all Gram-negative bacteria [1, 2]. They are primarily composed of outer membrane proteins, phospholipids, and lipopolysaccharides (LPSs), and are filled with periplasmic and cytoplasmic components such as peptidoglycan, proteins, nucleic acids, quorum sensing (QS) signals, and metal ions in the vesicle lumen [3–5]. Initially considered byproducts of bacterial cell lysis, OMVs are now known to be part of a unique bacterial secretion pathway termed type 0 secretion system (T0SS) [6]. Compared to classic secretion systems, the OMV-dependent T0SS delivers a diverse range of biologically active molecules in high concentrations, transports cargo long distances in a protected manner, and provides a mechanism for delivering a concentrated bolus of cargos to remote cells (bacterial or mammalian), thus eliminating the need for direct cell-to-cell contact [1, 5–7]. As a unique and versatile secretion system, OMVs are involved in multiple biological processes including cell-to-cell communication [8, 9], nutrition acquisition [10], horizontal gene transfer (HGT) [11], bacterial competition [12], stress tolerance [13], biofilm formation [14], antibiotic resistance [15], phage infection [16], and virulence [17, 18]. In addition, OMVs have shown great potential as vaccine platform [19, 20] and drug delivery vehicles for cancer therapy [21, 22].

In order to effectively transfer materials and transmit signals, OMVs must participate in specific interactions and eventually fuse with their target cells. Although the mechanisms through which OMVs deliver toxins to mammalian cells have been well-documented [23, 24], the molecular details of cargo delivery to bacterial cells remain largely unknown. While it is clear that secreted OMVs interact and fuse with microbial cells for cargo delivery [25–28], only one example based on ligand receptors for specific interactions between OMVs and bacterial cells has been reported in *Pseudomonas aeruginosa* [29]. The opportunistic pathogen *P. aeruginosa* packages the iron-chelating *Pseudomonas* quinolone signal (PQS) into OMVs for trafficking. To recognize OMVs, *P*. *aeruginosa* secretes the PQS-binding protein TseF, through the type VI secretion system H3 (H3-T6SS). Secreted TseF recognizes and associates with OMVs by interacting with PQS in OMVs, and facilitates the recruitment of OMVs to bacterial cells by interacting with the cell surface receptors FptA or OprF. The direct interaction between OMVs and recipient cells allows iron and PQS to be transported into the cell through an unknown mechanism. However, this model is limited because the PQS signal is only produced by *P*. *aeruginosa* and related species.

Recent studies have shown that OMVs can mediate cargo delivery between different species in microbial communities [9, 30–32]. For example, the OMVs produced by *Myxococcus xanthus*, which contain active proteases, phosphatases, hydrolases and secondary metabolites, are able to kill *Escherichia coli* cells by fusing with their outer membranes [31]. Similarly, OMVs from *Acinetobacter baylyi* were found to transfer DNA to *E. coli*, and other *A. baylyi* cells, via membrane fusion [32]. Moreover, OMVs play important roles in the delivery of hydrophobic QS signals between cells, which is achieved via target cell fusion [9]. While the interspecific sharing of OMVs in microbial communities is universal, the mechanisms by which bacteria recognize and recruit OMVs among different species remain unknown.

*Cupriavidus necator* JMP134 (formerly known as *Ralstonia eutropha* JMP134) is a versatile aromatic pollutant degrader belonging to the family Burkholderiales [33]. Although two T6SS gene clusters have been identified in the *C. necator* JMP134 genome, none have been experimentally characterized. In this study, we identified a unique LPS-binding effector, Reut_A1725 (hereafter referred to as TeoL, T6SS effector for recruitment of OMVs via LPS). TeoL is secreted by the Fur (ferric uptake regulator) regulated T6SS1 in *C*. *necator*, which recognizes OMVs derived from various bacterial species through interactions with LPS. TeoL tethers OMVs to the recipient cell surface by interacting with outer membrane receptors CubA and CstR. This LPS-based mechanism allows bacterial cells to use OMVs derived from different species to gain a competitive advantage over other cells in terms of iron acquisition, interbacterial competition, stress resistance, and HGT.

## Materials and methods

### Bacterial strains, plasmids, primers, and growth conditions

*Cupriavidus necator* strains (Table S1) were grown at 30 °C in Nutrient broth (NB) or in M9 minimal medium. *Pseudomonas aeruginosa* and *Yersinia pseudotuberculosis* strains (Table S1) were grown at 37 °C in tryptic soy broth (TSB), and 30 °C in Yersinia-Luria-Bertani (YLB) broth (1% tryptone, 0.5% yeast extract, 0.5% NaCl), respectively. Antibiotics were added at the following concentrations: ampicillin, 100 μg ml^−1^; kanamycin, 50 μg ml^−1^; gentamicin, 10 μg ml^−1^; nalidixic acid, 20 μg ml^−1^; tetracycline, 5 μg ml^−1^ for *Y. pseudotuberculosis*, 20 μg ml^−1^ for *C. necator*, 200 μg ml^−1^ for *P. aeruginosa*.

### Determination of intracellular ion contents

Intracellular ion contents were determined as described previously [34]. Briefly, cells were grown in M9 medium until stationary phase. After cells were collected and washed with M9 medium twice, the pellets weight was measured, resuspended in Bugbuster solution (Novagen, Madison, WI) and incubated on a rotating mixer for 16 h. Total protein for each sample was measured by using NanoDrop ND-1000 spectrophotometer (NanoDrop Technologies) and diluted ten-fold in 2% molecular grade nitric acid. Samples were further analyzed by inductively coupled plasma mass spectrometry (ICP-MS) (Varian 802-MS), and the results were corrected using the appropriate buffers for reference and dilution factors.

### OMV isolation, purification, and quantification

OMVs were isolated, purified and quantified as described [28, 29]. All OMVs were extracted from iron rich medium (NB for *C. necator*, TSB for *P. aeruginosa*, and YLB for *Y. pseudotuberculosis*). Briefly, to obtain OMVs without bacterial cells, overnight batch culture was centrifuged for 20 min at 6000 × *g*, 4 °C. The supernatant was filtered through 0.45 and 0.22 µm vacuum filter, respectively, to thoroughly remove remaining bacteria. The resulting filtrate was ultracentrifuged for 1 h at 200,000 × *g* at 4 °C using an angle rotor (70 Ti, Beckman Coulter, USA) and the pellets were washed twice with phosphate-buffered saline (PBS), which were subsequently resuspended in 50 mM HEPES-0.85% NaCl. For purification, crude OMV samples were adjusted to 1 ml of 45% (w/v) iodixanol (OptiPrep; Sigma-Aldrich) in HEPES-NaCl, transferred to the bottom of ultracentrifuge tubes, and layered with iodixanol-HEPES-NaCl (2 ml of 40, 35, 30, 25, and 20%). The samples were ultracentrifuged for 4 h at 150,000 × *g* at 4 °C using a swing rotor (SW40 Ti, Beckman Coulter, USA). Then, 1 ml fractions were collected from each gradient and detected by SDS-PAGE. The fraction containing OMV was ultracentrifuged for 1 h at 200,000 × *g* at 4 °C using an angle rotor and resuspended in HEPES-NaCl. For quantification, the protein concentration and the phospholipid concentration of the OMV were measured using previously reported methods [28, 35, 36], with bovine serum albumin and L-α-phosphatidylethanolamine as a reference standard, respectively.

### OMV association assay

Purified OMVs were fluorescently labeled with fluorescein isothiocyanate (FITC, Sigma-Aldrich) by incubation with 1 mg ml^−1^ FITC in 0.1 M sodium bicarbonate (pH 9.0) stirred for 1 h at 25 °C on a rotator [37]. The free dye was removed from the labeled OMVs by washing twice with PBS (200,000 × *g*, 1 h). Relevant strains were labeled by introducing a plasmid expressing mCherry (pME6032-*mCherry*), and late exponential phase bacterial cells were washed three times with PBS and incubated with FITC-labeled OMVs (30 µg ml^−1^ of phospholipids) for 4 h at 30 °C. After incubation, bacterial cells associated with FITC-labeled OMVs were washed with PBS three times. Washed cells were detected by confocal microscope and the percentages of cells exhibited the fluorescence of both mCherry and FITC (indicating the direct association of OMVs with bacterial cells) were quantified. Confocal microscopy was performed using a high-speed laser scanning confocal microscope (Andor Revolution WD, UK) with a ×100 oil immersion objective, and the images were processed using the ImageJ software.

### LPS-binding assay

The interaction between TeoL and LPS was performed with the pull-down assay by coupling of LPS to CNBr-activated Sepharose 4 Fast Flow gel (GE Healthcare, Piscataway, NJ) according to manufacturer’s specified protocol. Briefly, the preactivated gel was suspended in 1 mM HCl for 30 min to allow the gel to swell. After washed with 15 gel volumes of cold 1 mM HCl, 5 mg ml^−1^ LPS dissolved in coupling buffer (pH 8.3) was added to washed gel and incubated at room temperature for 3–4 h. The coupled gel was washed and resuspended in 100 mM Tris-HCl, and unused activated sites were then blocked for 2–4 h at room temperature. The LPS-coupled gel was washed three times with alternating 50 mM Tris-HCl, 0.5 M NaCl, pH 8.5 and 50 mM glycine, 0.5 M NaCl, pH 3.5 buffers, and saved in 20% ethyl alcohol after washed with ddH_2_O. To verify the interactions between LPS and TeoL, 0.04 mg GST-TeoL or GST were incubated with 100 μl LPS-coupled gel in 1 ml binding buffer for 4 h at 4 °C. After incubation, the gel was washed three times with TEN buffer, and retained proteins were detected by immunoblot with anti-GST antibody after SDS-PAGE.

### OMV-mediated gene transfer

Experiment for OMV-mediated gene transfer was performed as described [28]. Briefly, *C*. *necator* Δ*teoL* mutant harboring pBBR1MCS-2 (Km^R^) was grown in NB medium until the stationary phase, and plasmid-containing OMVs were extracted from the supernatant, treated with DNase I in reaction buffer (40 mM Tris-HCl, 10 mM NaCl, 6 mM MgCl_2_, 1 mM CaCl_2_, pH 7.9) to degrade external DNA surrounding OMVs, and washed with PBS by ultracentrifugation. DNase I treated OMVs (30 µg ml^−1^ of phospholipids) were then mixed with relevant *C. necator* strains (~2.5 × 10^3^ cells ml^−1^) in M9 medium. After incubation for 4 h at 30 °C, the cells were washed with M9 and spread on NB agar plates containing kanamycin, and the transfer of plasmid DNA was examined by counting the colony forming units (CFUs). As a control experiment, naked plasmid DNA, which was extracted from Δ*2Fe*(pBBR1MCS-2), was added to the cell suspension (final DNA concentration was 10 ng ml^−1^), and the possibility of natural transformation was examined by CFU counting. pBBR1MCS-2 concentration in OMVs was examined as described [28] by quantitative PCR analysis with primer pair KanR-F/KanR-R (Table S2).

### Statistical analysis

All experiments were performed at least in triplicate and repeated on two different occasions. Data are expressed as mean values ± SD. Differences between frequencies were assessed by the Student’s *t* test (bilateral and unpaired). Statistical analysis of results was conducted with GraphPad Prism version 8.2 (GraphPad software Inc; San Diego, CA, USA), using a *p* value of <0.05 as statistically significant.

Additional methods are described in Supplementary materials and methods.

## Results

### The Fur-regulated T6SS1 plays an important role in iron acquisition in *C*. *necator*

**Fig. 1:**
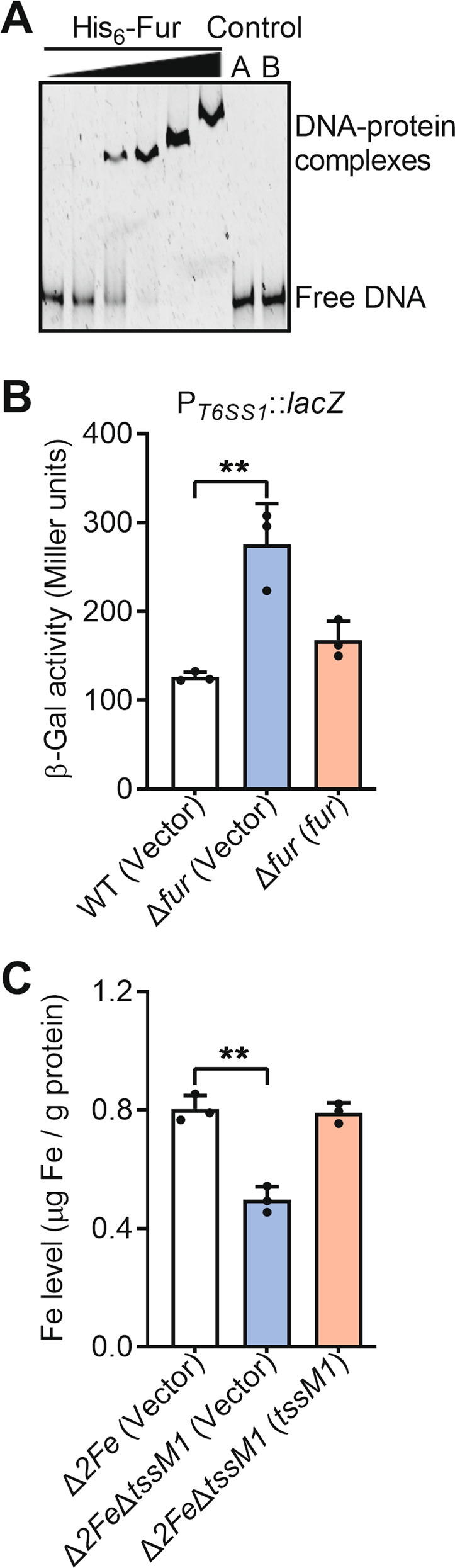
Regulation of T6SS1 expression by Fur. **A** The interactions between His_6_-Fur and the T6SS1 promoter examined by EMSA. Increasing amounts of Fur (0, 0.03, 0.06, 0.13, 0.25, and 1.0 μM) and 10 nM DNA fragments were used in the assay. A 500 bp unrelated DNA fragment (Control A) and 1 µM BSA (Control B) were included in the assay as negative controls. **B** Fur represses the expression of T6SS1. β-galactosidase activities of T6SS1 promoter from chromosomal *lacZ* fusions in relevant *C*. *necator* strains were measured. **C** Iron uptake requires T6SS1. Stationary-phase *C*. *necator* strains were washed twice with M9 medium. Iron associated with indicated bacterial cells were measured with ICP-MS. The vector corresponds to the plasmid pBBR1MCS-5 (**B**) and pBBR1MCS-2 (**C**), respectively. Data are represented as mean values ± SD of three biological replicates, each with three technical replicates. **p < 0.01.

To explore the function of T6SS1 (Reut_A1713 to Reut_A1733) in *C*. *necator* (Fig. S1A), we analyzed the T6SS1 promoter and identified a Fur binding site (AGAAATA) upstream of gene *reut_A1733*. This Fur binding site was highly similar to the Fur-box reported in *E. coli* [38], with a probability score of 2.25 (out of a maximum score = 2.45) (Fig. S1B), which was calculated by applying the position weight matrix to a sequence [39]. Incubation of the T6SS1 promoter probe with purified Fur protein led to decreased mobility of the probe in the electrophoretic mobility shift assay, suggesting a direct interaction between Fur and the T6SS1 promoter (Fig. 1A). To further determine the function of Fur on the expression of T6SS1, a single-copy *P*_*T6SS1*_*::lacZ* fusion reporter was introduced into the chromosomes of *C*. *necator* wild-type (WT), Δ*fur* deletion mutant, and the Δ*fur*(*fur*) complementary strain. Compared to WT, the *P*_*T6SS1*_*::lacZ* promoter activity was significantly increased in the Δ*fur* mutant (about 2.2-fold), and this increase could be restored by introducing the complementary plasmid pBBR1MCS-5-*fur* (Fig. 1B). Similar results were obtained by analyzing the expression of T6SS1 core component genes (*hcp1*, *clpV1*, *vgrG1*, and *tssM1*) with qRT-PCR (Fig. S1C). These results demonstrate that the expression of T6SS1 in *C*. *necator* is directly repressed by Fur, the master regulator of genes involved in iron homeostasis in many prokaryotes [40, 41].

To examine whether *C*. *necator* T6SS1 plays a role in iron homeostasis, we measured the intracellular iron contents of relevant strains in M9 medium using ICP-MS, and found no difference between the WT and T6SS1 mutant (Δ*tssM1*) (Fig. S2A). We speculated that the iron transport capacity of T6SS1 was masked by other major iron transport systems in *C. necator*, such as the cupriabactin siderophore iron transport system [34], and the FeoABC ferrous iron transport system [42]. As expected, deletion of *tssM1* in Δ*cubE*Δ*feoB* (hereafter referred to as Δ*2Fe*), a double mutant defected in both cupriabactin and FeoABC iron transport systems, significantly reduced intracellular iron level. However, the defect of the Δ*2Fe*Δ*tssM1* mutant in iron accumulation was fully restored by complementation of *tssM1* (Fig. 1C). By contrast, the accumulation of other metal ions (zinc, sodium, magnesium) was not affected by the deletion of *tssM1* in the Δ*2Fe* mutant (Fig. S2B). These results demonstrate that the *C*. *necator* T6SS1 is directly regulated by ferric uptake regulator Fur and is involved in iron acquisition.

### T6SS1 effector TeoL contributes to acquisition of iron from OMVs

**Fig. 2:**
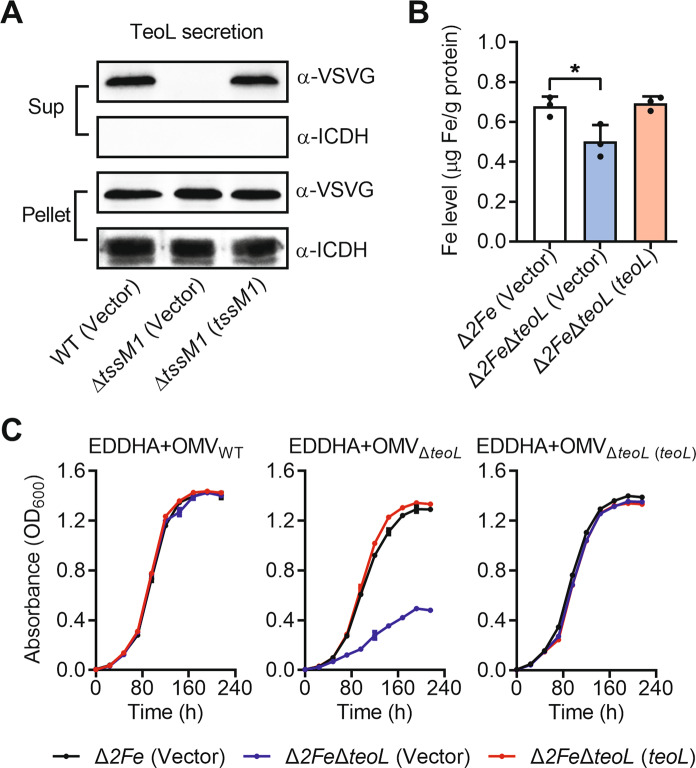
TeoL is a substrate of T6SS1 and contributes to iron acquisition by recruiting OMVs. **A** TeoL is a secreted substrate of T6SS1. Proteins in the culture supernatant of relevant *C*. *necator* strains expressing TeoL-VSVG were probed for VSVG by immunoblotting. The cytoplasmic protein ICDH (isocitrate dehydrogenase) was used as a loading control and lysis control for the pellet (Pellet) and supernatant (Sup) fractions. **B** TeoL is involved in iron acquisition. Stationary-phase *C*. *necator* strains were washed twice with M9 medium. Iron associated with indicated bacterial cells were measured with ICP-MS. **C** TeoL is required for *C*. *necator* uptake of iron from OMVs in iron-deficient media. The growth of the indicated bacterial strains was assessed in M9 medium containing EDDHA (5.5 μM) and OMVs (20 µg ml^−1^ of phospholipids) prepared from *C*. *necator* WT, Δ*teoL*, and Δ*teoL*(*teoL*), respectively. Cell growth was monitored by measuring optical density at 600 nm (OD_600_). The pBBR1MCS-2 plasmid was used as the vector for complementation. Data are represented as mean values ± SD of three biological replicates, each of which was performed in three technical replicates. *p < 0.05.

Lin et al. [29] reported that *P*. *aeruginosa* T6SS is involved in iron uptake by recruiting OMVs through TseF, a PQS-binding effector. Downstream of *vgrG1* in the *C*. *necator* T6SS1 gene cluster, we also identified a putative T6SS effector (Reut_A1725, hereafter, TeoL). While significant amounts of TeoL could easily be detected in culture supernatant of WT, the secretion of TeoL was completely abolished in Δ*tssM1* (Fig. 2A), and almost completely abolished in the Δ*clpV1* and Δ*hcp1* mutants (Fig. S3A). Even the residual TeoL secretion was completely abolished in Δ*clpV1*Δ*clpV2* and Δ*hcp1*Δ*hcp2* double mutants defected in both T6SSs in *C*. *necator* (Fig. S3B). Moreover, the secretion defects of these T6SS mutants could be completely restored to WT levels by complementation of corresponding T6SS1 component genes (Figs. 2A and S3). These results demonstrate that TeoL is an effector protein mainly secreted by T6SS1, though limited substrate cross recognition among T6SS1 and T6SS2 existed.

To examine the role of TeoL in iron acquisition, we produced a Δ*2Fe*Δ*teoL* mutant that consisted of a *teoL* deletion in the Δ*2Fe* background. While the Δ*2Fe*Δ*teoL* mutant grew equally in M9 medium as the Δ*2Fe* mutant, its growth was severely impaired compared to the Δ*2Fe* mutant in the iron-depleted M9 medium that contained 4.0 µM of the iron chelator ethylenediamine-*N*,*N*′-bis(2-hydroxyphenylacetic acid) (EDDHA) (Fig. S4A). However, the growth defect of the Δ*2Fe*Δ*teoL* mutant was completely rescued by plasmid-borne expression of *teoL*, or by adding excessive Fe^3+^ (0.5 μM) to the iron-depleted medium (Fig. S4A). Moreover, the Δ*2Fe*Δ*teoL* mutant exhibited significantly reduced intracellular iron levels compared to the Δ*2Fe* mutant and the Δ*2Fe*Δ*teoL*(*teoL*) complemented strain (Fig. 2B), though the accumulation of other metal ions was not affected (Fig. S2C). These results suggest that TeoL is involved in iron acquisition. However, we were unable to detect interactions between TeoL and Fe^3^^+^ (Fig. S5), suggesting that TeoL may not directly sequester iron as in the case of metal-binding T6SS effectors for metal ions transportation [43, 44].

To examine whether TeoL is involved in iron utilization from OMVs, we determined the effects of OMVs on the growth of Δ*2Fe*Δ*teoL* in iron-depleted M9 medium containing 5.5 μM EDDHA. As shown in Fig. S4B, both Δ*2Fe* and Δ*2Fe*Δ*teoL*(*teoL*) strains, but not Δ*2Fe*Δ*teoL*, exhibited increased growth with adding OMVs purified from distantly related Gram-negative bacteria, *P*. *aeruginosa* PAO1 and *Yersinia pseudotuberculosis* YPIII. Unexpectedly, the growth of Δ*2Fe*Δ*teoL* also increased following adding OMVs purified from the *C*. *necator* WT and Δ*teoL*(*teoL*) complemented strain (Fig. 2C). However, the adding of OMVs purified from the *C*. *necator* Δ*teoL* mutant had no effect on increasing Δ*2Fe*Δ*teoL* growth (Fig. 2C). These results demonstrate that TeoL plays crucial roles in acquiring iron derived from OMVs.

### TeoL is required for OMV recruitment in *C*. *necator*

**Fig. 3:**
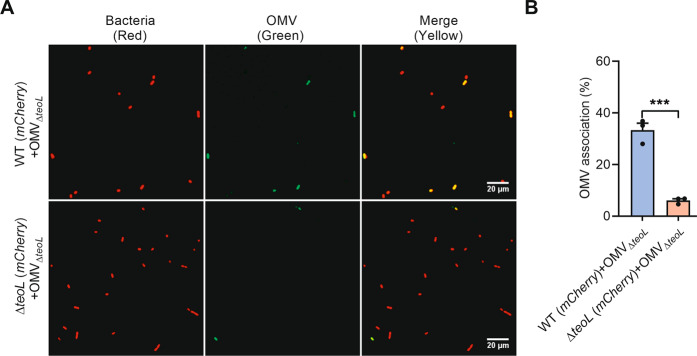
TeoL contributes to OMV recruitment. **A**, **B** The mCherry-labeled relevant *C*. *necator* strains were washed three times with PBS and incubated with FITC-labeled OMVs (30 µg ml^−1^ of phospholipids) derived from *C*. *necator* Δ*teoL* mutant for 4 h at 30 °C. After washed with PBS, the association between OMVs and the cells were observed by confocal microscopy (**A**). The percentages of cells that exhibited both mCherry and FITC fluorescence were quantified (**B**). The pictures were taken and processed using ImageJ software. Data are represented as mean values ± SD of three biological replicates, each with three technical replicates. ***p < 0.001.

The involvement of TeoL in acquiring iron derived from OMVs prompted us to further explore the role of TeoL in OMV recruitment. Thus, we incubated mCherry-labeled *C. necator* WT and Δ*teoL* mutant cells with FITC-labeled OMVs derived from the Δ*teoL* mutant. After 4 h of incubation, cells were washed and imaged with confocal microscopy and the percentages of cells exhibiting both mCherry and FITC fluorescence were quantified to measure the direct association between OMVs and bacterial cells. Although 33.3% of WT cells exhibited both mCherry and FITC fluorescence after incubation with fluorescent OMVs derived from Δ*teoL* mutant, the percentage of co-localized Δ*teoL* mutant cells decreased to 6.1% following incubation with OMVs derived from the Δ*teoL* mutant (Fig. 3), indicating that TeoL is involved in OMV recruitment.

To gain further insight into the role of TeoL in OMV recruitment, the interaction between TeoL and OMVs was examined using an assay based on glutathione-S-transferase (GST) pull-down. First, we introduced a plasmid expressing the OMV marker OmpW [45] tagged with the VSVG epitope into the Δ*teoL* mutant of *C*. *necator*. OmpW-VSVG containing OMVs purified from this strain were incubated with glutathione beads coated with GST-TeoL or GST, respectively, and OMVs captured on the glutathione beads were detected by immunoblot after SDS-PAGE using an anti-VSVG antibody for detecting the OmpW marker. As shown in Fig. S6A, capture of OmpW-VSVG containing OMVs was observed for the GST-TeoL fusion protein but not for the GST protein or beads-only control. This indicated that GST-TeoL directly interacts with OMVs prepared from *C*. *necator*. Interestingly, OMVs prepared from *P*. *aeruginosa* and *Y*. *pseudotuberculosis* showed the same binding results with GST-TeoL (Fig. S6A), suggesting that the interaction between TeoL and OMVs is not species-specific.

The interaction between TeoL and OMVs prompted us to further predict that secreted TeoL may associate with OMVs. Indeed, TeoL-VSVG was detected in OMVs purified from Δ*teoL* mutant expressing the *teoL-vsvg* fusion protein. Similarly, the OMV marker OmpW tagged with VSVG was also present in OMVs purified from the Δ*teoL* mutant expressing this fusion protein. By contrast, the VgrG1-VSVG protein, a core component of T6SS1, was not detectably associated with OMVs as predicted (Fig. S6B). These results suggest that TeoL directly associates with OMVs after secretion. We therefore concluded that TeoL contributes to OMV recruitment via direct interaction.

### TeoL recruits OMVs through binding LPS

**Fig. 4:**
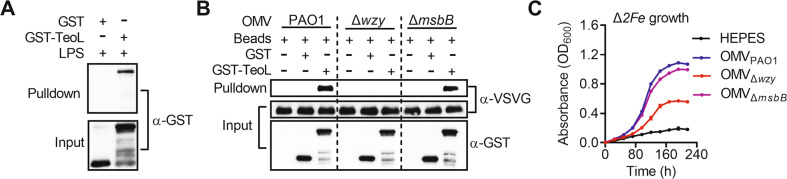
TeoL recruits OMVs through binding LPS. **A** TeoL interacts with LPS. LPS immobilized on Sepharose beads was incubated with GST-TeoL and the formation of the LPS-TeoL complex was detected by immunoblot. GST was used as a negative control. **B** The O-antigen region of LPS is required for TeoL-mediated OMV recruitment. GST or GST-TeoL was incubated with OMVs purified from OprF-VSVG expressing *P. aeruginosa* PAO1, Δ*wzy*, and Δ*msbB*, respectively. The formation of the TeoL-OMV complex was captured by glutathione beads and detected by immunoblot with anti-VSVG antibody. **C** The O-antigen region of LPS is required for acquisition of iron from OMVs. The growth of the *C*. *necator* Δ*2Fe* mutant was assessed in M9 medium containing EDDHA (5.5 μM) and OMVs (20 µg ml^−1^ of phospholipids) prepared from *P. aeruginosa* PAO1, Δ*msbB*, and Δ*wzy*, respectively. Cell growth was monitored by measuring optical density at 600 nm (OD_600_). Data are represented as mean values ± SD of three biological replicates, each of which was performed in three technical replicates.

Above results suggest that TeoL targets OMVs for recruitment to the bacterial cell, yet the OMV component that determines TeoL targeting is unknown. Because LPS is the main component of OMVs, we investigated whether LPS was necessary and sufficient to link TeoL with OMVs. As shown in Fig. 4A, LPS immobilized on Sepharose beads efficiently precipitated the GST-TeoL protein but not GST, indicating direct binding between TeoL and LPS. The disassociation constant (*K*_*d*_) between TeoL and LPS was 0.58 μM (Fig. S7A) as measured using isothermal titration calorimetry (ITC), comparable to that of CD4, a well-known LPS-binding protein [46]. The negative control GST did not bind LPS, as detected under the same binding conditions (Fig. S7A).

LPS is composed of three distinct domains: the lipid A moiety, the core oligosaccharides, and the O-antigen [47]. To determine which part of LPS is required for TeoL binding, the interactions between TeoL and different rough (R) forms of LPS with varying polysaccharide chains (Ra, Rc, and Rd) were investigated. As shown in Fig. S7B, no binding was detected between TeoL and lipid A in ITC analyses, and compared to LPS and lipid A, rough LPS showed an intermediate binding affinity. Moreover, the longest R form tested (Ra) showed the strongest binding affinity (*K*_*d*_ = 14.3 μM) and the shortest R form tested (Rd) showed the weakest binding affinity (*K*_*d*_ = 105.6 μM). These results suggest that the O-antigen region in LPS may directly interact with TeoL.

To further verify the roles of lipid A and O-antigen in OMV recruitment, we produced the *P*. *aeruginosa* PAO1 lipid A biosynthesis mutant (Δ*msbB*) [48] and O-antigen biosynthesis mutant (Δ*wzy*) [49, 50]. OMVs prepared from PAO1 and Δ*msbB* showed efficient TeoL binding while OMVs prepared from the Δ*wzy* mutant failed to interact with TeoL (Fig. 4B). Moreover, the growth of Δ*2Fe* mutant under iron-depleted medium was efficiently increased by addition of OMVs prepared from WT and Δ*msbB* mutant, while adding OMVs prepared from Δ*wzy* mutant showed a very weak effect (Fig. 4C).

Since both OMVs and bacterial outer membranes contain LPSs, our next challenge was to uncover how TeoL distinguishes between LPSs on OMVs and LPSs on bacterial outer membranes. We speculated that TeoL might exhibit higher binding affinity to OMV-associated LPSs, enabling OMV-specific binding. Consistent with our hypothesis, ITC analysis revealed that TeoL exhibited a 4.7-fold higher affinity to LPSs purified from OMVs compared to those purified from bacterial cells (Fig. S8). Taken together, these results indicate that TeoL recognizes LPS, particularly LPS derived from OMVs, through binding to its O-antigen component.

### TeoL guides OMV recruitment by binding to outer membrane receptors CubA and CstR

**Fig. 5:**
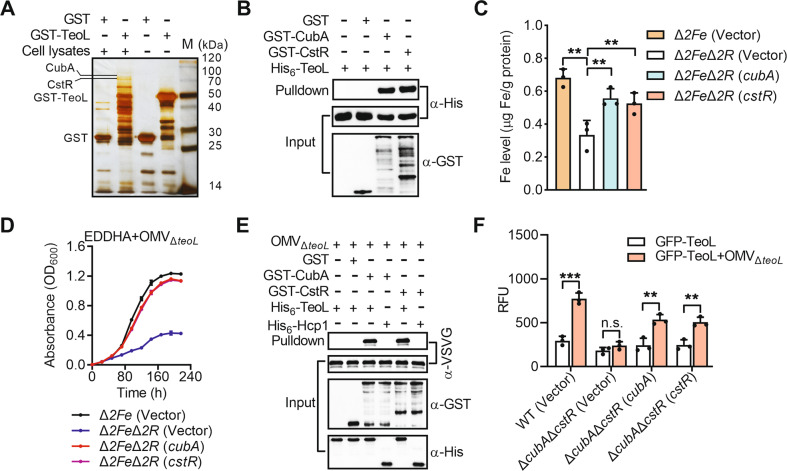
CubA and CstR are required for TeoL-mediated OMV recruitment. **A** CubA and CstR were retained by agarose beads coated with GST-TeoL. Total cell lysates of *C*. *necator* were incubated with beads coated with GST or GST-TeoL. After removing unbound proteins, the proteins retained were resolved by SDS-PAGE followed by silver staining. **B** Direct binding of TeoL to CubA and CstR. His_6_-TeoL was incubated with GST, GST-CubA, or GST-CstR. Protein complexes were captured by glutathione beads and were detected by Western blotting. **C** CubA and CstR are involved in iron acquisition in *C*. *necator*. Stationary phase *C*. *necator* strains were collected and washed twice with M9. Iron associated with bacterial cells was measured by ICP-MS. **D** CubA and CstR are required for obtaining iron derived from OMVs. The growth of the indicated bacterial strains was assessed in M9 medium containing EDDHA (5.5 μM) and Δ*teoL* OMVs (20 µg ml^−1^ of phospholipids). Cell growth was monitored by measuring optical density at 600 nm (OD_600_). **E** TeoL bridges the interactions between OMVs and CubA or CstR. GST, GST-CubA, or GST-CstR were incubated with OMVs prepared from the OmpW-VSVG expressing Δ*teoL* mutant in the presence or absence of His_6_-TeoL. The formed protein-OMV complexes were captured by glutathione beads and detected by Western blotting with anti-VSVG antibody. His_6_-Hcp1 was used as a control. **F** The formation of the TeoL-OMV complex is a prerequisite for TeoL binding to the bacterial cell surface. GFP-TeoL (preincubated with or without Δ*teoL* OMVs) was incubated with *C*. *necator* WT, Δ*cubA*Δ*cstR* double mutant, and Δ*cubA*Δ*cstR*(*cubA*) and Δ*cubA*Δ*cstR*(*cstR*) complemented strains in 1 ml PBS for 3 h at 30 °C. After removing unbound GFP-TeoL protein with centrifugation, cell pellets were resuspended in 1 ml PBS and GFP-TeoL associated to bacterial cells was determined at the recommended wavelength (Ex/Em: 490/510 nm) using a fluorescence spectrometer. Data are represented as mean values ± SD of three biological replicates, each with three technical replicates. ***p < 0.001; **p < 0.01; ns not significant.

Despite the finding that TeoL recognizes OMVs through LPS, the mechanism of OMV recruitment by the bacterial cell is still unclear. We hypothesized that TeoL may direct OMVs to the bacterial cell surface by interacting with specific outer membrane receptors. To identify possible binding receptors, we performed affinity chromatography with GST-TeoL-coated beads against total cell lysates of *C*. *necator* WT. After washing with TEN buffer, proteins retained by GST-TeoL were visualized with silver staining after SDS-PAGE (Fig. 5A). Two specific bands around 80 kDa were identified by mass spectrometric analysis. These were identified as the cupriabactin siderophore receptor CubA (Reut_B3686) [34], and the catecholate siderophore receptor Reut_B4659 (hereafter refer to as CstR). Both CubA and CstR are siderophore-related TonB-dependent outer membrane receptor proteins. The specific interactions between TeoL and CubA or CstR were validated by in vitro binding assays with purified proteins (Fig. 5B). To determine the role of these receptors in iron acquisition, we constructed Δ*2Fe*Δ*cubA*Δ*cstR* (hereafter referred to as Δ*2Fe*Δ*2R*) mutant in which *cubA* and *cstR* were deleted in the background of strain Δ*2Fe*. While the Δ*2Fe*Δ*2R* mutant showed severely reduced intracellular iron accumulation in M9 medium, this reduction was rescued by complementation with either *cubA* or *cstR* alone, thus confirming their roles in iron acquisition (Fig. 5C).

To further determine the roles of these receptors in OMV recruitment, we compared the growth of the Δ*2Fe*Δ*2R* mutant with Δ*2Fe* in an iron-depleted medium supplemented with OMVs prepared from the Δ*teoL* mutant. The growth of the Δ*2Fe*Δ*2R* mutant was significantly lower compared to the Δ*2Fe* mutant, which was completely restored by introducing a plasmid expressing either *cubA* or *cstR* (Fig. 5D). Similar results were obtained by adding OMVs purified from *P*. *aeruginosa* PAO1 to the iron-depleted medium (Fig. S9A). The role of CubA and CstR receptors in OMV recruitment was further confirmed by observing the direct association between mCherry-labeled bacterial cells and FITC-labeled OMVs purified from the Δ*teoL* mutant (Fig. S10). These results suggest that CubA and CstR are involved in TeoL-mediated OMV recruitment and iron acquisition.

To garner additional insight into CubA and CstR functions in TeoL-mediated OMV recruitment, we incubated GST-tagged receptors with *C*. *necator* Δ*teoL* OMVs labeled with OmpW-VSVG in the presence or absence of the TeoL protein, respectively. After precipitation with glutathione beads, receptor-OMV complexes retained on the glutathione beads were resolved by SDS-PAGE and detected by immunoblot with an anti-VSVG antibody for detecting the OmpW marker. Although both CubA and CstR specifically bound to OMVs, the binding was strictly dependent on the presence of TeoL (Fig. 5E). Similar results were obtained with OprF-VSVG marked OMVs [51] purified from *P. aeruginosa* (Fig. S9B). These results suggest that during OMV recruitment, the role of TeoL is to tether iron-containing OMVs to specific receptors on the cell surface.

This conclusion was further supported by directly measuring the binding of GFP-TeoL proteins (preincubated with or without Δ*teoL* OMVs) to *C*. *necator* WT, the Δ*cubA*Δ*cstR* double mutant, and the Δ*cubA*Δ*cstR*(*cubA*) and Δ*cubA*Δ*cstR*(*cstR*) complemented strains using a fluorescence spectrometer (Fig. 5F). While the GFP-TeoL protein alone exhibited weak binding affinities to all strains even *C*. *necator* WT, preincubation of the GFP-TeoL protein with Δ*teoL* OMVs greatly improved its affinity to *C*. *necator* WT and the Δ*cubA*Δ*cstR*(*cubA*) and Δ*cubA*Δ*cstR*(*cstR*) complemented strains. However, preincubation with Δ*teoL* OMVs did not improve the affinity of GFP-TeoL to the Δ*cubA*Δ*cstR* double mutant. The finding that preincubation with OMVs enhanced the binding affinities of TeoL to bacterial cells further corroborated its role in tethering OMVs to the bacterial cell surface through recognition of the outer membrane CubA/CstR receptors.

We then speculated that secreted TeoL may exhibit a binding preference for OMVs over bacterial cells. To validate this hypothesis, we incubated GFP-TeoL proteins with Δ*teoL* cells or OMVs containing equal amounts of LPS (30 µg ml^−1^ of phospholipids), respectively, and the amounts of GFP-TeoL associated with OMVs or bacterial cells were quantified using a fluorescence spectrometer after removing unbound GFP-TeoL proteins in the supernatant with ultracentrifugation. As predicted, GFP-TeoL showed stronger associations with Δ*teoL* OMVs than Δ*teoL* cells (Fig. S11), consistent with our finding that TeoL exhibited higher affinities to LPSs purified from OMVs than to those purified from bacterial cells (Fig. S8). Taken together, these results suggest that once secreted, the TeoL effector protein selectively binds to OMVs first, then brings the iron-containing OMVs to the bacterial cell surface by interacting with the CubA/CstR outer membrane receptors.

### TeoL-mediated OMV recruitment is crucial for exploitation competition, oxidative stress resistance, and horizontal gene transfer

**Fig. 6:**
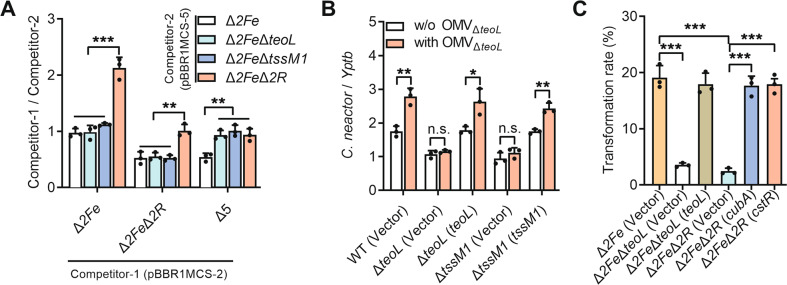
TeoL-mediated OMV recruitment is important for exploitation competition and horizontal gene transfer. **A**, **B** TeoL-mediated OMV recruitment contributes to exploitation competition. Intraspecies growth competition between the indicated competitor 1 strains (Containing pBBR1MCS-2, Km^R^) and competitor 2 strains (Containing pBBR1MCS-5, Gm^R^) after co-incubated for 12 h at 30 °C in M9 medium containing OMVs (20 µg ml^−1^ of phospholipids) prepared from the Δ*teoL* mutant. The competitive index result is calculated as the final CFUs ratio (Competitor 1/Competitor 2) divided by the initial ratio (**A**). Interspecies growth competition between relevant *C*. *necator* strains and *Y. pseudotuberculosis* YPIII in M9 medium containing 0.5 µM EDDHA, with or without Δ*teoL* OMVs (20 μg ml^−1^ of phospholipids). The CFUs ratio of the relevant *C. necator* strains versus *Y. pseudotuberculosis* was calculated by determining the CFUs before (initial) and after (final) growth competition (**B**). **C** TeoL-mediated OMV recruitment contributes to HGT. OMVs were extracted from the stationary phase culture of *C*. *necator* Δ*teoL* mutant harboring pBBR1MCS-2 (Km^R^). DNase I-treated OMVs (30 μg ml^−1^ of phospholipids) were incubated with relevant *C*. *necator* strains at 30 °C. The transformation rate was calculated by counting the CFUs on agar plates containing kanamycin. Data are represented as mean values ± SD of three biological replicates each with three technical replicates. ***p < 0.001; **p < 0.01; *p < 0.05; ns not significant.

T6SSs enhance bacterial survival by delivering “anti-bacterial” toxins [52, 53] or by enhancing its ability to acquire essential micronutrients such as manganese and zinc during exploitative competition (such as consuming nutrients from the milieu) [43, 44, 54, 55]. The finding that TeoL/T6SS1 is required for iron acquisition from OMVs suggests that they play a role in mediating exploitation competition. To test this hypothesis, we performed intraspecies growth competition assays between *C*. *necator* strains with differed capabilities in TeoL secretion and OMVs recruitment, in M9 medium containing Δ*teoL* OMVs (20 µg ml^−1^ of phospholipids). As shown in Fig. 6A, the Δ*2Fe* strain showed increased growth compared to the Δ*2Fe*Δ*2R* strain, because although both strains can secrete TeoL, only the Δ*2Fe* strain can recruit OMVs with CubA/CstR receptors. Δ*2Fe*Δ*2R* did not show competition advantage over Δ*2Fe*Δ*teoL* and Δ*2Fe*Δ*tssM1*, which cannot secrete TeoL but can recruit OMVs with CubA/CstR receptors. These results suggest that bacteria that possess functional OMV receptors can use TeoL-associated OMVs produced by other bacteria, regardless of their ability to secrete TeoL. Consistent with this conclusion, the Δ*2Fe*Δ*2R* strain displayed a severe growth disadvantage when competing with not only Δ*2Fe*, but also with the Δ*2Fe*Δ*teoL* and Δ*2Fe*Δ*tssM1* strains. One possible explanation is that the Δ*2Fe*Δ*2R* strain, which cannot recruit OMVs, can still produce TeoL-associated OMVs to support the growth of the Δ*2Fe*Δ*teoL* and Δ*2Fe*Δ*tssM1* strains. As expected, the Δ*2Fe*Δ*teoL* and Δ*2Fe*Δ*tssM1* strains displayed no growth advantage over the Δ*2Fe*Δ*2R*Δ*teoL* (hereafter referred to as Δ*5*) strain, which cannot produce TeoL-associated OMVs. The role of TeoL/T6SS1 in mediating exploitative competition was further confirmed by interspecies growth competition assays between *C*. *necator* strains and *Y*. *pseudotuberculosis*. As shown in Fig. 6B, while the *C*. *necator* WT showed increased growth compared to *Y*. *pseudotuberculosis* in the absence of Δ*teoL* OMVs (1.8-fold), it was highly competitive against the *Y*. *pseudotuberculosis* competitor in the presence of Δ*teoL* OMVs (2.8-fold). However, the competitive advantage of *C*. *necator* WT was largely abolished in Δ*teoL* and Δ*tssM1* mutants, and such deficits could be rescued by complementation with corresponding genes.

Similar to T6SSs reported in *Y*. *pseudotuberculosis* [44] and *Burkholderia thailandensis* [43], the *C*. *necator* T6SS1 also contributed to defense against oxidative stress (Fig. S12A). Indeed, deleting *teoL* alone was sufficient to decrease resistance to H_2_O_2_ in *C*. *necator* (Fig. S12A), suggesting that OMVs recruited by TeoL is important for resistance to oxidative stress. To determine the functions of OMVs in resisting oxidative stress, we used the Δ*5* mutant, which has deficits in iron acquisition, OMV recruitment, and TeoL production. The survival rates of Δ*5* and its corresponding single gene complemented strains were determined following exposure to H_2_O_2_ for 25 min, in the absence or presence of OMVs purified from WT, Δ*teoL*, and Δ*teoL*(*teoL*) strains, respectively. While adding all three types of OMVs significantly increased the survival rates of the WT strain, adding these OMVs had no effect on the Δ*5* mutant, indicating that the capability to obtain OMVs is crucial for resisting oxidative stress (Fig. S12B). Moreover, adding OMVs purified from WT and Δ*teoL*(*teoL*) complementary strains, but not the Δ*teoL* mutant strain, substantially improved the survival rates of Δ*5* complemented with OMV receptor genes *cubA* or *cstR*, but not *teoL*. These results suggest that the presence of TeoL (no matter provided by the bacteria cells themselves or by added OMVs) and one of the receptors allowed the bacteria to obtain OMVs for resisting oxidative stress.

OMVs are also known to be involved in HGT [11]. To determine whether the TeoL/T6SS1-mediated OMV recruitment pathway contributes to HGT, we evaluated plasmid DNA transfer mediated by OMVs. The *C*. *necator* Δ*teoL* mutant harboring pBBR1MCS-2 (Km^R^) was grown in NB medium until the stationary phase, and plasmid-containing OMVs were extracted from the supernatant. About 1.87 × 10^5^ copies of pBBR1MCS-2 plasmid were detected to be associated with OMVs in 1 ml of the culture supernatant (7.30 × 10^5^ and 5.43 × 10^5^ copies ml^−1^ in the supernatant before and after removing OMVs through ultracentrifugation, respectively). After degrading the external DNA surrounding OMVs by DNase I treatment, about 1.22 × 10^5^ copies of pBBR1MCS-2 plasmid in the OMVs from 1 ml culture supernatant (equivalent to 1.48 × 10^7^ copies mg^−1^ OMV phospholipids) were detected. When relevant *C*. *necator* cells (~2.5 × 10^3^ cells ml^−1^) were incubated with an excessive amount of OMVs (30 μg ml^−1^ phospholipids), more than 19.1% of Δ*2Fe* transformants were obtained on selective plates containing kanamycin after incubation with Δ*teoL* OMVs for 4 h at 30 °C, suggesting that the plasmid contained in the OMVs was transferred to bacterial cells. However, the Δ*2Fe*Δ*teoL* and Δ*2Fe*Δ*2R* mutants preincubated with Δ*teoL* OMVs showed a remarkable decrease in transformation efficiency (3.6% and 2.4%, respectively), and the decreased transformation efficiency could be substantially restored by complementation (Fig. 6C). Notably, natural transformation did not occur in *C. necator* when naked plasmid DNA (10 ng ml^−1^) extracted from Δ*teoL*(pBBR1MCS-2) was directly added to bacterial cell suspension.

Together, these results demonstrate that the TeoL/T6SS1-mediated OMV recruitment pathway is crucial for obtaining cargos loaded in OMVs, thus performing pleiotropic physiological functions.

## Discussion

OMVs have garnered much attention for their broad functions in intercellular interactions and potential uses as vaccine and drug delivery vectors. However, research has only just begun to shed light on the mechanism by which OMVs interact with bacterial cells. In this study, we revealed that *C*. *necator* T6SS1 secretes an LPS-binding protein, TeoL, to recognize and actively recruit OMVs for cargo acquisition. Because LPS is one of the most abundant components of OMVs, this LPS-based mechanism allows recipient cells to use OMVs derived from various species as public goods. We also showed that *C*. *necator* T6SS1 recruits OMVs to the bacterial cell surface by interacting with the outer membrane receptors CubA and CstR. This ligand-receptor interaction-based OMV recruitment pathway provides recipient cells with the opportunity to use OMVs produced by various bacterial species, and may represent a general mechanism applicable to other Gram-negative bacteria.

The role of LPS in mediating OMV recruitment was supported by several lines of evidence. First, we confirmed through in vitro binding assays that TeoL and LPS bind directly (Fig. 4A). Next, we revealed that TeoL directly binds to the O-antigen region, which constitutes the outermost structural region of LPS, and may be the first component to contact recipient cells due to its length (up to 40 nm) (Fig. S7B) [56]. Moreover, unlike OMVs prepared from the WT strain and the lipid A biosynthesis mutant Δ*msbB*, OMVs prepared from the O-antigen biosynthesis mutant Δ*wzy* failed to interact with TeoL (Fig. 4B). While the growth of the Δ*2Fe* mutant in an iron-depleted medium was efficiently increased by adding OMVs prepared from the WT and Δ*msbB* mutant strains, adding OMVs prepared from the Δ*wzy* mutant had very weak effect on increasing the growth of the Δ*2Fe* mutant (Fig. 4C).

Both OMVs and bacterial outer membrane contain LPS. Thus, one important question is why TeoL prefers to bind on LPS from the OMVs rather than LPS from intact cells. Several previous studies reported that bacterial cells and OMVs have different LPS composition [26, 57]. Specifically, these data revealed that LPS isolated from bacterial cells is comprised of a mixture of rough and smooth A-band and B-band LPS and LPS isolated from OMVs contains only B-band LPS, resulting in different composition and arrangement between bacterial LPS and OMVs’ LPS. Moreover, Schooling and Beveridge [14] reported that the low-density biofilm OMVs have more LPS and less protein than their planktonic counterparts. Remarkably, we found that TeoL binding to LPSs purified from OMVs was 4.7-fold stronger compared to LPSs purified from bacterial cells (Fig. S8), which enables secreted TeoL to preferentially bind to OMVs, as opposed to bacterial cells, in natural environments. Indeed, neither OMVs nor TeoL can be effectively recruited to bacterial cell surfaces until they form OMV-TeoL complexes (Fig. 5F).

Iron has long been considered one of the main cargos carried by OMVs because they are enriched in proteins involved in iron acquisition, such as FetA [58], IhtB [59], and TbpB [60]. OMVs from *P*. *aeruginosa* are enriched in the highly hydrophobic iron chelator, PQS, which enables bacteria to soak up iron from the environment [61]. In addition, membrane vesicles of *Dietzia* sp. DQ12-45-1b, a Gram-positive bacterium, participate in extracellular heme capture with heme-binding proteins, allowing the heme carried in MVs to be utilized by multiple related species [62]. Furthermore, iron deficit leads to increased OMV production in *Haemophilus influenzae* by downregulation of the Fur-regulated VacJ/Yrb ABC phospholipid transporter. VacJ/Yrb ABC is pivotal for OMV production as it regulates phosphorlipid accumulation in the outer membrane [63]. Although these findings suggest that OMVs can scavenge iron and deliver it to bacterial cells, this has only been verified in *P*. *aeruginosa*. Under iron-limited conditions, *P*. *aeruginosa* H3-T6SS secretes a PQS-binding effector TseF to recognize and recruit OMVs to the surface of bacterial cells for iron uptake [29]. Although this research revealed a novel OMV-dependent iron acquisition pathway, this species-specific mechanism fails to explain how OMV-iron can be shared in the bacterial community. The finding that *C. necator* T6SS1 secretes an LPS-binding protein (TeoL) to recruit OMVs for iron acquisition that allows bacterial cells to use OMVs produced by various species as iron sources provides the first general mechanism for OMVs as public goods. Interestingly, similar to the VacJ/Yrb ABC phospholipid transporter (responsible for OMV production) [63], both the *C*. *necator* T6SS1 and the *P*. *aeruginosa* H3-T6SS [29] are regulated by Fur and iron starvation (Figs. 1A, B and S1C), implicating that all these systems are required for iron acquisition. Together, these studies suggest a complex circuit for the OMV-mediated iron acquisition pathway, which involves OMV biogenesis, T6SS secretion, OMV recognition mediated by TeoL, and OMV recruitment mediated by outer membrane siderophore receptors.

The T6SS-based OMV recruitment pathway also provides new insight into T6SS functionality. T6SS is a phage tail-like transmembrane machinery used by many Gram-negative bacteria to kill competing microbes by injecting toxic effectors into adjacent cells through direct contact [64, 65]. Recently, T6SSs were also reported as giving bacteria a competitive advantage by improving their abilities to acquire essential nutrients (such as zinc and manganese) [43, 44]. Indeed, the T6SS1/TeoL-mediated OMV-dependent iron acquisition pathway is also involved in contact-independent exploitative competition under iron-limited conditions (Fig. 6A, B). To the best of our knowledge, this study is the first to report the role of T6SS in bacterial competition through the use of OMVs. It further supports the idea that T6SS gives bacteria a competitive advantage and acts as evolutionary factors that shape the composition of the microbial community [43, 66–68].

Recently, T6SS was also reported to play a role in HGT. In *Vibrio cholerae*, T6SS is part of the competence regulon and is co-induced with genes involved in natural competence by the competence regulators TfoX and QstR on chitinous surfaces. T6SS-dependent killing of neighboring non-immune cells leads to their lysis, and the released DNA can be accessed by the competent predator cells for HGT [69]. Similarly, the naturally competent *A. baylyi* ADP1 was found to use T6SS to lyse cells and thereby enhance HGT [70, 71]. Interestingly, here we found that the *C*. *necator* T6SS promoted HGT by facilitating DNA acquisition from OMVs (Fig. 6C). This finding not only reveals the links between T6SS, OMVs, and HGT, but also provides a new perspective for understanding the roles of T6SS in promoting HGT and the spread of antibiotic resistance genes.

**Fig. 7:**
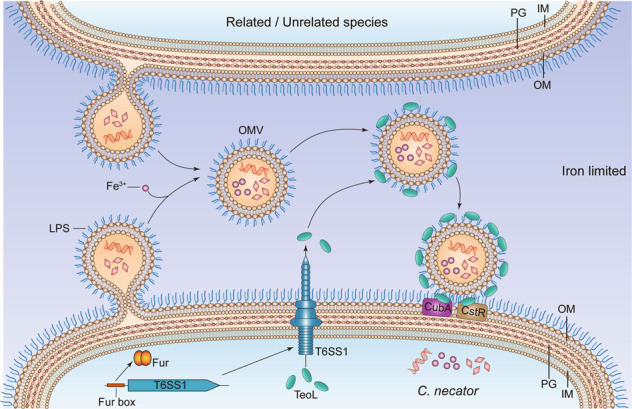
Model of the LPS-dependent interspecific OMV recruitment mechanism. Under iron-deficient conditions, the Fur-repressed T6SS1 gene cluster is de-repressed and secretes the LPS-binding effector TeoL. After secretion by T6SS1, TeoL binds to LPS on the outer leaflet of OMVs (derived from related/unrelated species) and remains at the surface and further binds CubA and CstR on the surface of recipient cells, which tethers OMVs to the recipient cells. Cargos in the OMVs are delivered into recipient cells by a yet unidentified mechanism. OM outer membrane, IM inner membrane, PG peptidoglycan.

Based on our results, we propose a novel and potentially universal mechanism for OMV recruitment by bacterial cells, which may be widely applicable to Gram-negative bacteria (Fig. 7). Under iron-deficient conditions, the Fur-repressed T6SS1 gene cluster is de-repressed and secretes the LPS-binding effector TeoL. After secretion by T6SS1, TeoL binds to LPS on the outer leaflet of OMVs and remains at the surface. TeoL can further bind CubA and CstR on the surface of recipient cells, which tethers OMVs to the recipient cells. The intimate contact with recipient cells allows OMVs to deliver cargo with diverse chemical compositions and perform various physiological functions such as nutrition acquisition, stress tolerance, bacterial competition, and HGT. Because LPS is a common component of OMVs in Gram-negative bacteria, this model provides a mechanism for mediating bacterial interactions where OMVs from different species can support maintenance of other Gram-negative species in the microbial community. The presence of T6SS related TeoL-like proteins in a diverse array of bacteria (Fig. S13) suggests that this OMV recruitment mechanism is widely distributed. Thus, the LPS-based model of OMV recruitment could be applicable to a large number of Gram-negative bacteria.

## Supplementary information

Supplementary information
