# Supplementary material for: T6SS secretes an LPS-binding effector to recruit OMVs for exploitative competition and horizontal gene transfer
Source: ISME J. 2021 Aug 25;16(2):500–10. doi: 10.1038/s41396-021-01093-8 (PMC8776902; doi:10.1038/s41396-021-01093-8)
Supplement: Supplementary file 1 — Supplementary information [file 41396_2021_1093_MOESM1_ESM.pdf]

T6SS secretes an LPS-binding effector to recruit OMVs for exploitative competition and horizontal gene transfer

Supplementary Information

This PDF file includes:

Supplementary Materials and Methods

Supplementary Figures 1-13

Supplementary Tables 1-2

Supplementary References

Supplementary Materials and Methods

Plasmid construction

To construct the knock-out plasmid for deletion of *teoL* (*Reut_A1725*) in *C. necator*, the 833 bp upstream fragment and the 774 bp downstream fragment of *teoL* were amplified using the primer pairs *teoL*-1F-BglII/*teoL*-1R and *teoL*-2F/*teoL*-2R-SalI (Table S2), respectively. The upstream and downstream PCR fragments were ligated by overlap PCR, and the resulting PCR product was digested with BglII/HindIII and inserted into the BamHI/HindIII sites of the suicide vector pK18*mobsacB* to produce pK18-Δ*teoL*. The knock-out plasmids pK18-Δ*tssM1* (*Reut_A1715*), pK18-Δ*clpV1* (*Reut_A1727*), pK18-Δ*hcp1* (*Reut_A1731*), pK18-Δ*feoB* (*Reut_A5429*) and pK18-Δ*cstR* (*Reut_B4659*) were constructed in a similar manner. The Gm resistance gene was amplified from plasmid pBBR1MCS-5 using the primer pairs Gm-F-BglII/Gm-R-BglII (Table S2) and inserted into the pK18 to generate pK18-Gm, then the pK18-Gm-Δ*msbB* (*PA3242*) and pK18-Gm-Δ*wzy* (*PA3154*) were constructed as that to generate pK18-Δ*teoL*. To complement the Δ*teoL* mutant, primers *teoL*-F-EcoRI/*teoL*-R-BamHI (Table S2) were used to amplify the *teoL* gene from genomic DNA. The PCR product of *teoL* was digested with EcoRI/BamHI and inserted into the EcoRI/BamHI sites of pBBR1MCS-2 and pBBR1MCS-5, respectively, to produce the complementation plasmid pBBR1MCS-2-*teoL* and pBBR1MCS-5-*teoL.* The complementary plasmids pBBR1MCS-2-*tssM1*, pBBR1MCS-2-*clpV1*, pBBR1MCS-2-*hcp1* and pBBR1MCS-2-*cstR* were constructed similarly.

Plasmid pME6032-*teoL*-*vsvg* was constructed for protein secretion assay. Briefly, primers *teoL*-F-EcoRI/*teoL*-R-*vsvg*-BglII (Table S2) were used to amplify the *teoL* gene, and the PCR product of *teoL*-*vsvg* was inserted into the EcoRI/BglII site of pME6032 to generate

pME6032-*teoL*-*vsvg*. Similarly, the plasmids pME6032-*ompW* (*reut_A3378*)-*vsvg*, pME6032-*vgrG1* (*reut_A1726*)-*vsvg*, pME6032-*ompW* (*ypk_2049*)-*vsvg*, pME6032-*oprF* (*PA1777*)-*vsvg* pME6032-*teoL*-*his*, pME6032-*vgrG1*-*his* and pME6032-*ompW* (*reut_A3378*)-*his* were constructed. For constructing expression plasmids, the genes encoding *C. necator* TeoL, Hcp1, CubA and CstR were amplified by PCR. The obtained DNA fragments were digested and cloned into similar digested pGEX-6p-1 and pET28a, yielding corresponding plasmid derivatives. To construct the *lacZ* fusion reporter pK18-*T6SS1p::lacZ*, primers *lacZ*-F-XbaI/*lacZ*-R-SphI (Table S2) were used to amplify the *lacZ* gene fragment from DH5α genomic DNA. The PCR product of *lacZY* was digested with XbaI/SphI and inserted into the XbaI/SphI sites of pK18*mobsacB* to generate pK18-*lacZ*. The primers P_{T6SS1P}-F-BamHI/P_{T6SS1}-R-XbaI (Table S2) were used to amplify the 606 bp T6SS1 promoter fragment from *C. necator* genomic DNA. The PCR product was digested with BamHI/XbaI and inserted into similarly digested pK18-*lacZ* to produce pK18-*T6SS1p::lacZ.* The integrity of the insert in all constructs was confirmed by DNA sequencing.

In-frame deletion, chromosomal fusion reporters and β-galactosidase assays

For constructing in-frame deletion mutants, the pK18*mobsacB* derivatives were transformed into relevant *C. necator* and *P. aeruginosa* strains through *E. coli* S17-1*λ pir*-mediated conjugational mating and screened as described [1, 2]. The resulting deletion mutants were verified by PCR and DNA sequencing. For overexpression or complementation, the pME6032 and pBBR1MCS derivatives were transformed into relevant strains by electroporation. The *lacZ* fusion reporter plasmid pK18-*T6SS1p::lacZ* was transformed into *E. coli* S17-1*λpir* and

mated with *C. necator* strains as described previously [1]. The *lacZ* fusion reporter strains were grown in NB medium at pH 7.0 under 30°C, and β-galactosidase activity was assayed using *o*-Nitrophenyl β-D-galactopyranoside (ONPG) as the substrate and expressed in Miller units [3]. All assays were performed in triplicate at least three times, and error bars represent standard deviations.

Recombinant protein purification, protein secretion and western blotting

His_6- and GST-tagged recombinant proteins were expressed and purified from *E. coli* as described [4]. The GST tag was removed by incubation with PreScission Protease (GE healthcare) for 16 h at 4°C. Secretion assays for TeoL were performed according to described methods [5]. In brief, relevant *C. necator* strains expressing TeoL-VSVG were grown to mid-exponential phase. 2 ml culture was taken and the pellet was resuspended in 100 μl SDS loading buffer to serve as total cell pellet sample. A total of 150 ml culture was centrifuged and the supernatant was filtered through a 0.22 μm filter (Millipore, MA, USA). The supernatant was filtered over a nitrocellulose filter (BA85, Whatman, Germany) three times to maximally absorb all secreted proteins in the supernatant. The nitrocellulose filter was soaked in 100 μl SDS loading buffer for 30 min at 55°C to recover secreted proteins and then boiled for 20 min. All samples were normalized to the OD_{600} of the culture and volume used in preparation. For western blotting analysis, samples were resolved by SDS-PAGE and transferred onto polyvinylidene difluoride (PVDF) membranes (Millipore). After blocking with 5% (w/v) BSA in TBST (50 mM Tris, 150 mM NaCl, 0.05% Tween 20, pH 7.4), membranes were incubated with the appropriate primary antibody: anti-VSVG, 1:10,000; anti-His, 1:1000;

anti-GST (Santa Cruz Biotechnology, USA), 1:1000; anti-ICDH [6], 1:6000. The membranes were washed and incubated with horseradish peroxidase-conjugated secondary antibodies (Shanghai Genomics) for 2 h. Signals were detected using the ECL plus kit (GE Healthcare, Piscataway, NJ) following the manufacturer's specified protocol.

Isothermal titration calorimetry (ITC)

The interactions between TeoL and LPS or Fe^{3+} were measured using isothermal titration calorimetry (ITC) at 25°C with a NANO-ITC 2G microcalorimeter (TA Instruments, USA) [7]. The LPS were extracted using LPS extraction kit (iNtRON Biotechnology) as reported [8] and quantized with LPS ELISA kit (Cloud Clone Corp), and the commercial LPS and its variant were purchased from Sigma-Aldrich. The proteins and chemicals examined were all prepared with ITC buffer (50 mM Tris, 150 mM NaCl, 15% [v/v] glycerol, pH 8.0). For detecting interactions between TeoL and LPS, 2.0 mg ml^{-1} LPS was filled into the syringe compartment (250 μl) while the protein solution (10 μM) was dispensed into the microcalorimetric cell (volume 950 μl). After a stable baseline was achieved the LPS titration was performed by a total of 25 injections of 10 μl into protein solutions until the protein sample was saturated with LPS. LPS variants with different lengths of polysaccharide core (LPS-Ra, Rc and Rd), and lipid A were also detected in the same manner. Blank titrations of the LPS solution into the dialysis buffer were performed to correct for the dilution heat of the LPS solution. As a negative control, LPS was titrated into the GST protein dispersion. For the binding assay between Fe^{3+} and protein, the apo-proteins were prepared by dialysis against 250 μM EDTA and 5 mM *o*-phenanthroline in 25 mM Tris-HCl (pH 7.4) for 6 h before dialysis in the ITC buffer

and 1.0 mM iron chloride was injected into 20 μM protein. Data reduction and analysis were performed with the Nano Analyze software (TA Instruments) fitting them to an independent binding model [9].

GST pull-down assay

The GST pull-down assay was performed as previously described with minor modifications [10]. For screening TeoL interaction partners in *C. necator*, stationary phase *C. necator* cells was lysed in Bugbuster solution (Novagen, Madison, WI) and incubation on a rotating mixer at a slow setting for 4 h. After being centrifuged, the cleared lysate was subsequently incubated with 0.1 mg purified GST-TeoL and 100 μl prewashed glutathione beads on a rotating mixer for 4 h at 4°C. After washed with TEN buffer (50 mM Tris-HCl, 10 mM EDTA, 150 mM NaCl, pH 8.0), the proteins associated with beads were solubilized with SDS loading buffer, separated by SDS-PAGE and detected by silver staining (Bio-Rad). Gel slices containing individual protein bands were excised, digested with trypsin and analyzed by matrix-assisted laser desorption/ionization/mass spectrometry (Voyager-DESTR, Applied Biosystems).

To determine protein interactions with purified proteins, 0.01 mg purified GST or GST-fusion proteins were incubated with 0.01 mg His-tagged proteins in TEN buffer for 2 h at 4°C. After adding 40 μl of prewashed glutathione beads, binding was allowed to proceed for another 2 h. The beads were then washed with TEN buffer containing 300-500 mM NaCl. Retained proteins were detected by immunoblotting after SDS–PAGE.

To determine the interactions between GST or GST-fusion proteins with OMVs, the

plasmids pME6032-*ompW* (*Reut_A3378*)-*vsvg*, pME6032-*ompW* (*YPK_2049*)-vsvg and pME6032-*oprF* (*PA1777*)-*vsvg* expressing the OMV marker OmpW (Reut_A3378) of *C. necator*, OmpW (YPK_2049) of *Y. pseudotuberculosis* and OprF (PA1777, OmpA-homolog) of *P. aeruginosa* tagged with the VSVG epitope was expressed in relevant strains, from which the OMVs were isolated and purified. 0.04 mg GST-fusion proteins were mixed with OMVs (10 µg of phospholipids) on a rotator at 4°C. For detection of the roles of TeoL in mediation of the interactions between GST tagged receptors and OMVs, 0.04 mg His-tagged TeoL was also included in the binding mixture. After adding 40 µl of prewashed glutathione beads, binding was allowed to proceed for another 2 h, and the beads associated with proteins were then washed with TEN buffer containing 300-500 mM NaCl. Potential protein-OMV complexes retained on the glutathione beads were detected by immunoblot with anti-VSVG antibody after SDS-PAGE.

Iron binding assay

Fe^{3+} binding activity of TeoL was also detected using the metal reconstitution assay as previously described [10]. Briefly, the resulting apo-TeoL protein (10 µM) was added to 20 µM Fe^{3+} and incubated on ice for 1 h, with Milli-Q water for preparing ions solution as the control. These solutions were dialyzed as mentioned above to remove unbound Fe^{3+}, and Fe^{3+} bound to the protein was analyzed using atomic absorption spectroscopy (ZEEnit 650P; Analytik Jena).

Protein association assay

To determine the association of TeoL with bacterial cells, relevant *C. necator* cells grown to the late exponential phase (OD_{600} = 1.5) were washed and diluted in PBS to an OD_{600} of 1.0, which were subsequently incubated with 20 μg of GFP-tagged TeoL (pre-incubated with or without Δ*teoL* OMVs (30 μg of phospholipids)) in 1 ml PBS for 3 h at 30°C. After removing unbound GFP-TeoL protein with centrifugation, cell pellets were resuspended in 1 ml PBS and GFP-TeoL associated to bacterial cells was determined at the recommended wavelength (Ex/Em: 490/510 nm) using a fluorescence spectrometer (SpectraMax M2, Molecular Devices, America). To compare the binding affinity of TeoL to OMVs and bacterial cells, 100 μg of GFP-TeoL protein was incubated with Δ*teoL* OMVs or Δ*teoL* cells containing equal amounts of LPS (150 μg of phospholipids) in 5 ml PBS, respectively. The unbound proteins and the proteins associated to bacterial cells or OMVs were separated from 1 ml mixture by ultracentrifugation (200,000 × *g*, 1 h, 4°C) at each time points. After resuspended in 1 ml PBS, the fluorescence of GFP-TeoL in supernatant, resuspended cell pellets and OMVs were determined using a fluorescence spectrometer at the recommended wavelength (Ex/Em: 490/510 nm), respectively, and the percentage of each component was calculated at each time points.

Sensitivity assay for oxidative agents

Sensitivity assay was performed as reported [10]. Briefly, mid-exponential phase *C. necator* strains grown in NB medium were collected, washed, and diluted 50-fold into M9 medium containing OMVs (20 μg ml^{-1} of phospholipids) or not, and treated with H_2O_2 (0.1 mM) at 30°C for 25 min. After treatment, the cultures were serially diluted and plated onto NB agar medium,

and colonies were counted after 36 h growth at 30°C. Percentage survival was calculated by dividing CFUs of treated cells by CFUs of control. All these assays were performed in triplicate at least three times.

Intraspecies and interspecies growth competition assays

Intraspecies competition and interspecies competition assay were performed as described previously [10]. For intraspecies competition, overnight-grown competitor 1 and competitor 2 strains were washed with M9 medium before mixing for competition. The competitor 1 and competitor 2 strains contain the kanamycin resistance plasmid pBBR1-MCS-2 and the gentamicin resistance plasmid pBBR1-MCS-5, respectively. The initial competitor 1/competitor 2 ratio was 1:1, and the bacteria mixture were incubated at 30°C with agitation for 12 h in liquid M9 medium (2 ml), in the presence or absence of OMVs (20 μg ml^{-1} of phospholipids). After competition, the competitor 1 and competitor 2 colonies were counted on NB plates supplemented with kanamycin or gentamicin, and changes in the competitor 1/competitor 2 ratios were determined. For interspecies competition, overnight cultures of relevant *C. necator* strains harboring plasmid pBBR1-MCS-2 (Km^R) and *Y. pseudotuberculosis* competitors containing plasmid pBBR1-MCS-5 (Gm^R) were washed three times with M9 medium, and then mixed in 2:1 (v/v) of relevant *C. necator* versus the competitor *Y. pseudotuberculosis*. To calculate the initial CFU ratio of relevant strains, 100 μl of the mixture was taken out, serially diluted, spread on NB and YLB plates containing different antibiotics, and incubated at 30°C for 24 h. For competition, the bacteria mixtures were incubated at 30°C with agitation in M9 medium containing EDDHA (0.5 μM), in the presence

or absence of OMVs (20 μg ml^{-1} of phospholipids). After 24 h, the mixture was serially diluted, spread on NB and YLB agar plates containing different antibiotics, and the final CFU ratio was determined. Data from all competitions were analyzed using the Student's t test.

Electrophoretic mobility shift assay (EMSA)

EMSA was performed as previously described [11]. Briefly, increasing concentrations of purified His_6-Fur (0, 0.03, 0.06, 0.13, 0.25 and 1 μM) were incubated with 10 nM T6SS1p DNA probes in reaction buffer (20 mM Tris-HCl, pH 7.4, 4 mM $MgCl_2$, 100 mM NaCl, 1 mM dithiothreitol, 10% glycerol), with a 500 bp unrelated fragment DNA (10 nM) and 1 μM BSA (Bovine Serum Albumin) as negative controls. After incubation for 20 min at room temperature, the binding reaction mixture was subjected to electrophoresis on a 6% native polyacrylamide gel, and the DNA probe was detected using SYBR Green.

Quantitative real-time PCR

Bacterial cells were harvested during the mid-exponential phase and RNAprep Pure Cell/Bacteria Kit was used to extract RNA. TransScript First-Strand cDNA Synthesis SuperMix and TransStart Green qPCR SuperMix (TransGen Biotech, Beijing, China) were used to reverse transcribe first-strand cDNA and perform quantitative real-time PCR (qRT-PCR), respectively. And the relative abundance of 16S rRNA was used as the internal standard. The copy number of the plasmid was determined using real-time PCR analysis. The kanamycin resistant gene in pBBR1MCS-2 was amplified and quantified using a primer pair KanR-F and KanR-R (Table S2). All samples were analyzed in triplicate, and the expression of target genes was calculated as relative fold values using the $2^{-\Delta\Delta Ct}$ method. All assays were

performed in triplicate at least three times, and error bars represent standard error of the mean.

Supplementary Figures

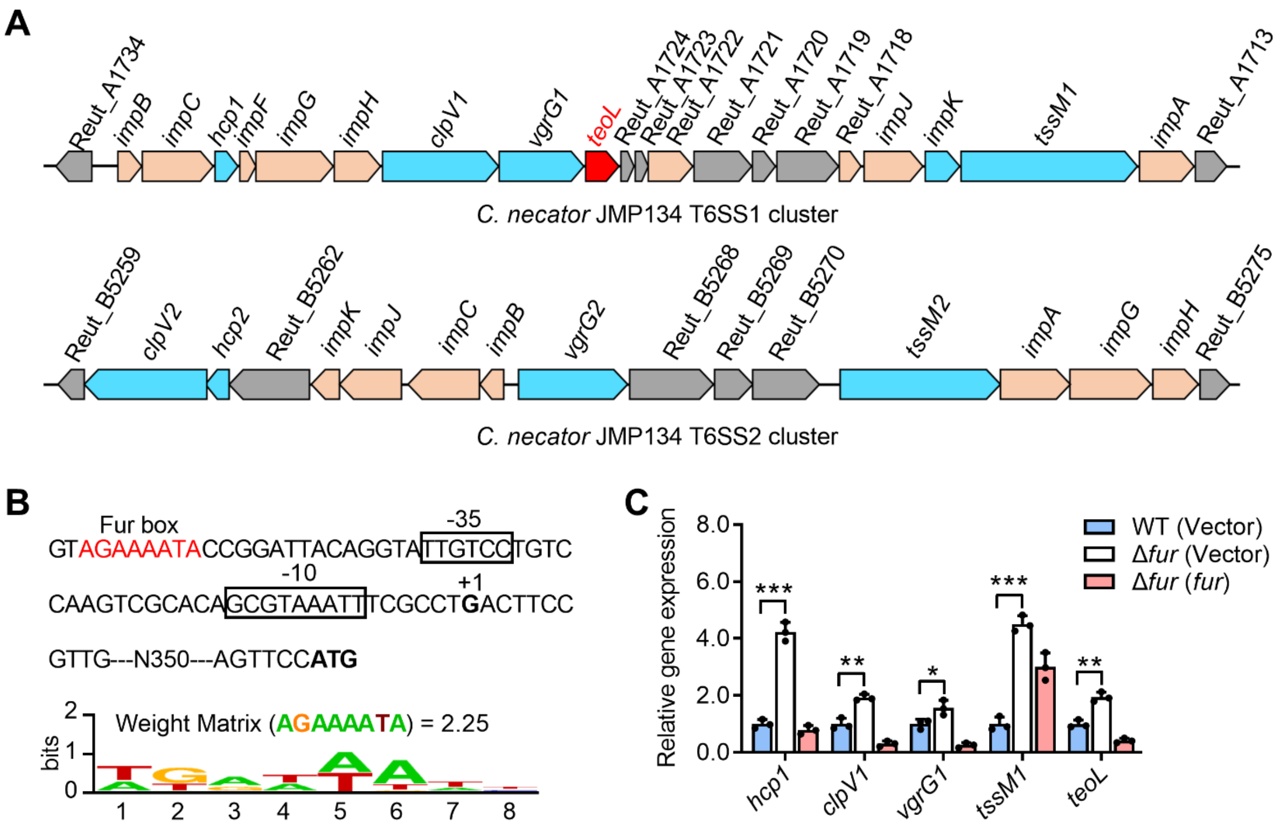

Fig. S1. Regulation of T6SS1 expression by Fur.
A. The organization of the *C. necator* T6SS1 and T6SS2 gene clusters. Structural genes are depicted as colored arrows that indicate transcriptional directions. **B.** Upstream *Reut_A1733* was T6SS1 promoter region and a putative Fur-binding site (AGAAAATA) was identified by the online software Virtual Footprint. Putative -35 and -10 elements of the T6SS1 promoter are outlined in the box. Letters represent position weight matrix based on *E. coli* K-12 consensus sequence for Fur binding, and *C. necator* Fur box sequence has a probability score of 2.25 (max score = 2.45), which is calculated by applying the position weight matrix to a sequence. The Y-axis represents relative nucleotide probability and the X-axis represents nucleotide position. **C.** Fur represses the expression of T6SS1. Gene expression of the major components of T6SS1 was measured using qRT-PCR. The vector corresponds to the pBBR1MCS-2 plasmid. Relative levels of transcripts are presented as mean values ± SD calculated from three sets of independent experiments. ***, $p < 0.001$; **, $p < 0.01$; *, $p < 0.05$.

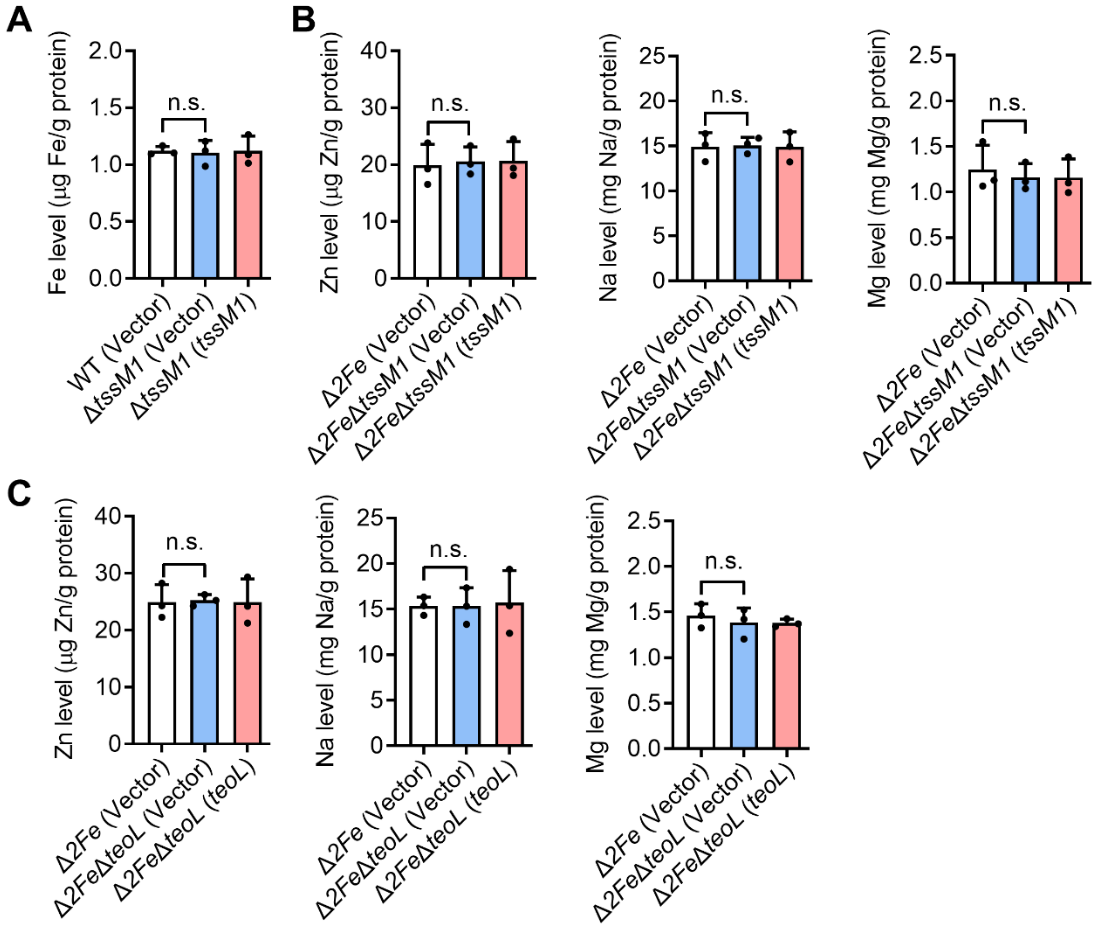

Fig. S2. T6SS1 and TeoL are not involved in Zn^{2+}, Na^{+} and Mg^{2+} accumulation in *C. necator*.

A-C. Determination of intracellular ion concentrations of *C. necator* relevant strains. Stationary phase *C. necator* strains were collected and washed twice with M9 medium, and metal ions associated with bacterial cells were measured by ICP-MS. The vector corresponds to the pBBR1MCS-2 plasmid. Data are represented as mean values ± SD of three biological replicates, each of which was performed in three technical replicates. n.s., not significant.

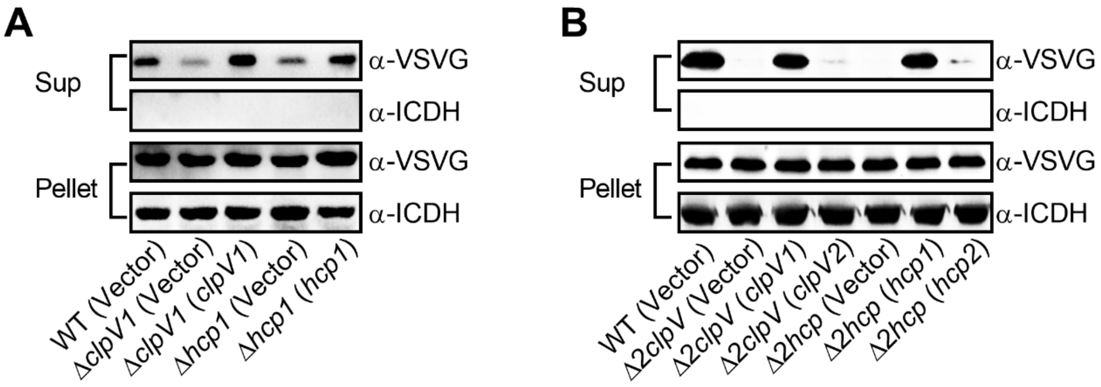

Fig. S3. TeoL is a secretion substrate of T6SS1.

A-B. Proteins in the culture supernatant of relevant *C. necator* strains expressing TeoL-VSVG were probed for VSVG by immunoblotting. The cytoplasmic protein ICDH was used as a loading control and lysis control for the pellet (Pellet) and supernatant (Sup) fractions.

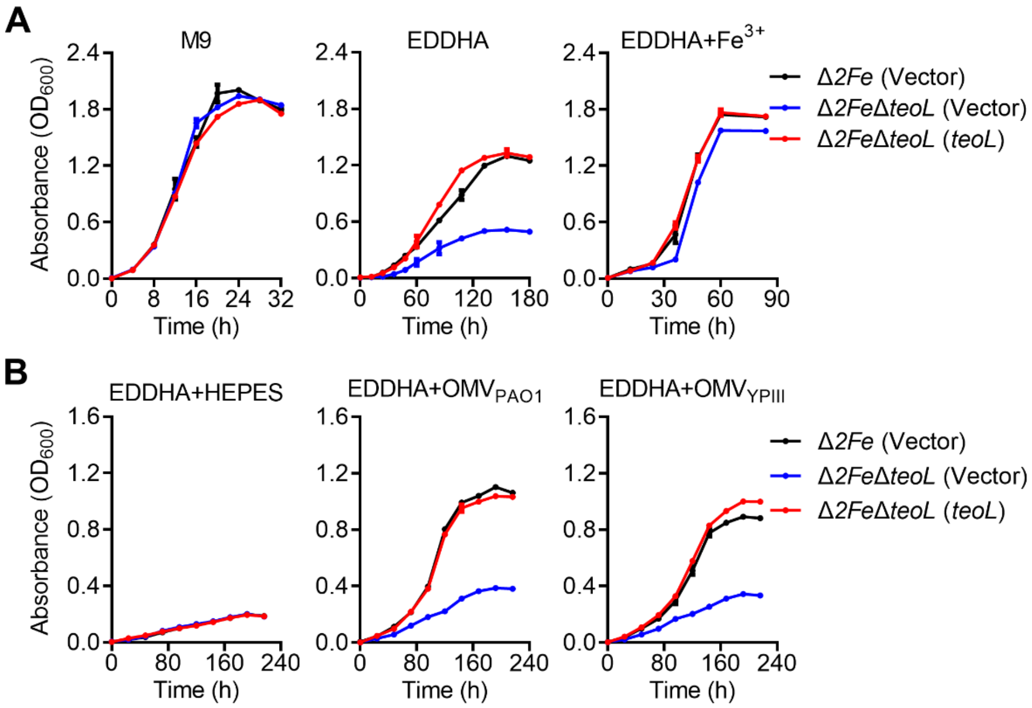

Fig. S4. TeoL is required for *C. necator* uptake of iron from OMVs in iron-deficient media. A-B. Relevant bacterial strains were grown until the stationary phase in NB medium and diluted to M9, M9 containing EDDHA (4.0 μM), EDDHA (4.0 μM) with Fe^{3+} (0.5 μM) (**A**) or EDDHA (5.5 μM) with OMVs (20 μg ml^{-1} of phospholipids) derived from strains (**B**). HEPES was used as a control. Cell growth was monitored by measuring optical density at 600 nm (OD_{600}). The vector corresponds to the pBBR1MCS-2 plasmid. Data are represented as mean values ± SD of three biological replicates, each of which was performed in three technical replicates.

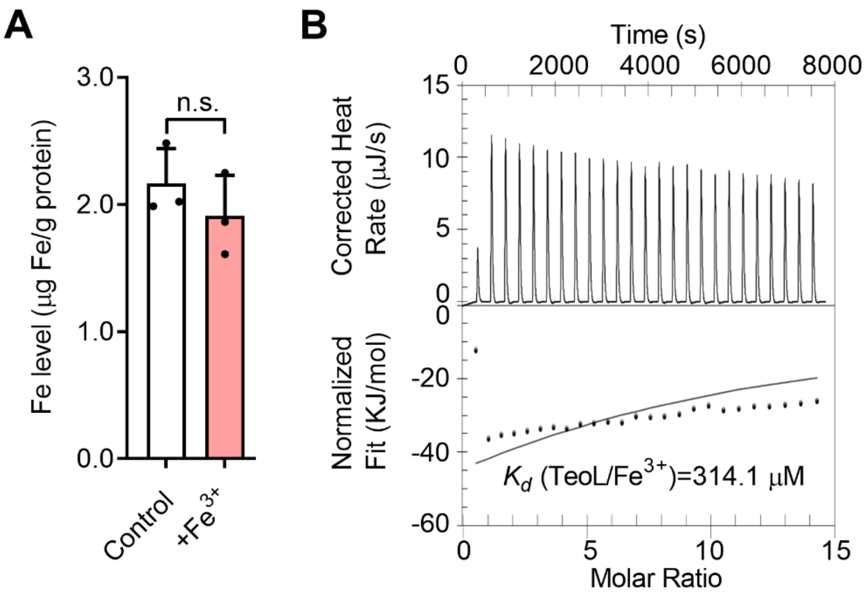

Fig. S5. TeoL doesn't bind iron.

A-B. The binding of iron by TeoL was detected by atomic absorption spectrometry (**A**) and ITC (**B**). Data are represented as mean values ± SD of three biological replicates, each of which was performed in three technical replicates. n.s., not significant.

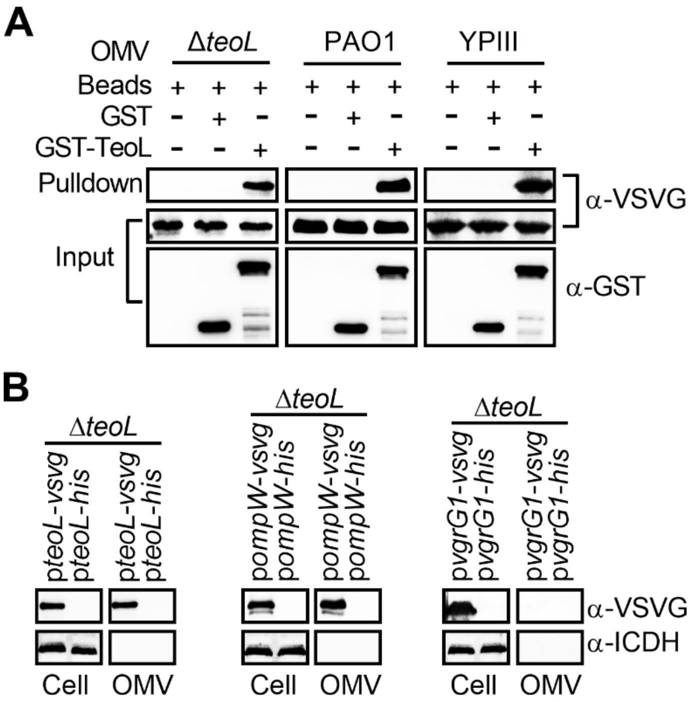

Fig. S6. TeoL contributes to OMV recruitment via direct interaction.

A. TeoL interacts with OMVs derived from different species. GST or GST-TeoL was incubated with OMVs prepared from OmpW-VSVG expressing Δ*teoL*, OprF-VSVG expressing *P. aeruginosa* PAO1, or OmpW-VSVG expressing *Y. pseudotuberculosis* YPIII, respectively. The protein–OMV complex formed was captured using glutathione beads and was detected by Western blotting. **B.** Secreted TeoL is associated with OMVs. OMVs prepared from the Δ*teoL* mutant expressing TeoL-VSVG, OmpW-VSVG, or VgrG1-VSVG and the proteins of interest were probed. The cytoplasmic protein ICDH was detected as a control.

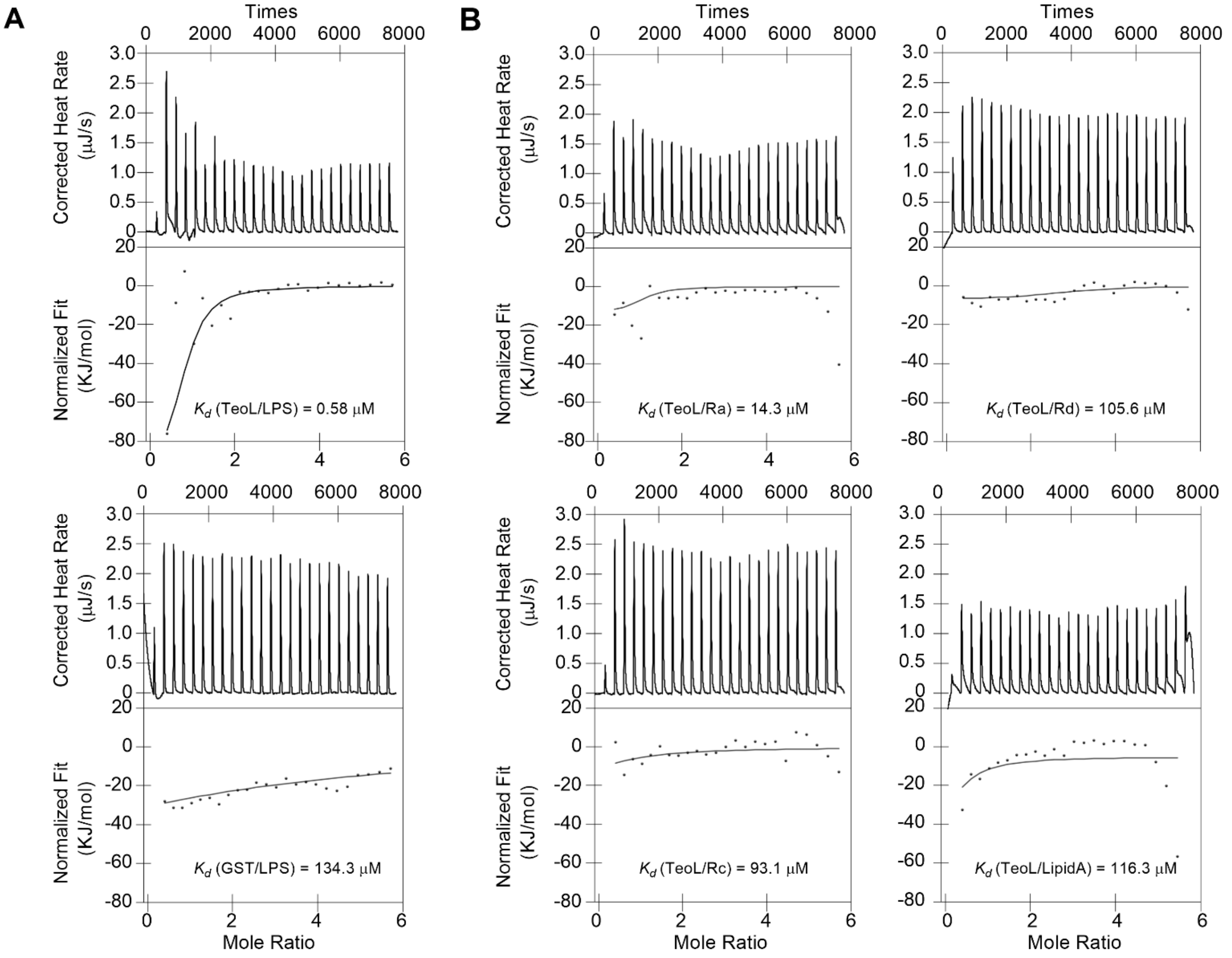

Fig. S7. TeoL interact with LPS through binding to its O-antigen component.

A-B. The interaction between TeoL and LPS (**A**) or different LPS variants (Ra, Rc, Rd and LipidA) (**B**) were determined by ITC, and GST was used as a control. Data were analyzed using the NanoAnalyze software.

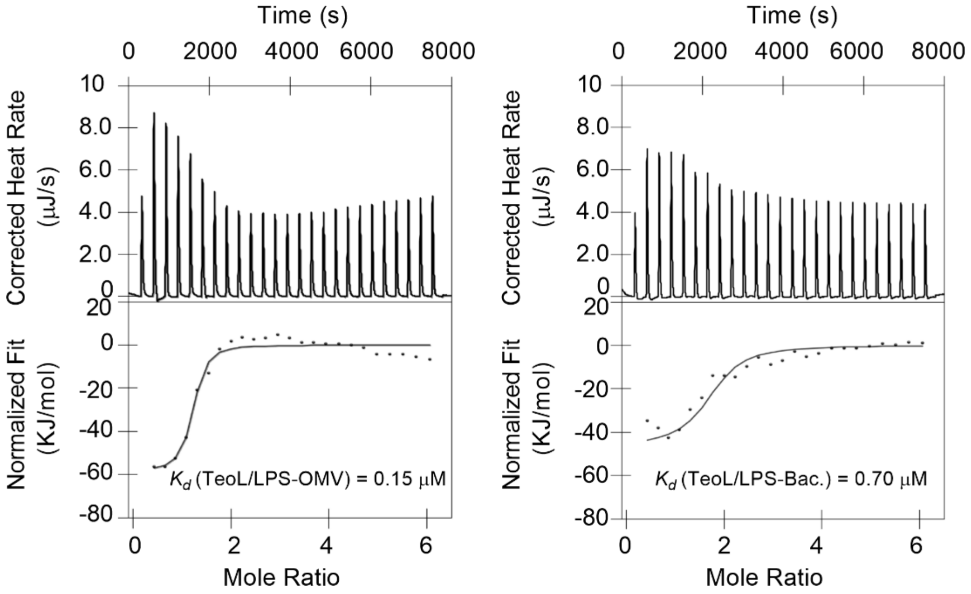

Fig. S8. TeoL prefers to bind LPS derived from OMVs.
The affinity between TeoL and LPS prepared from bacteria cells or OMVs, respectively, were determined by ITC. Data were analyzed using the NanoAnalyze software.

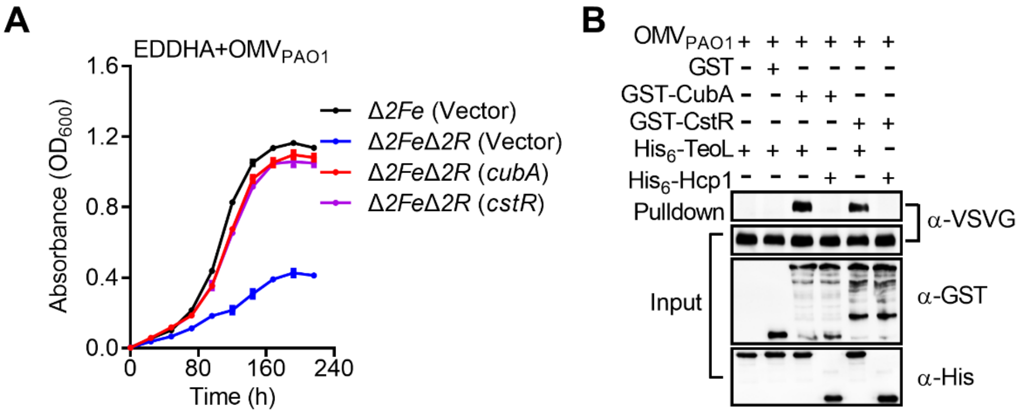

Fig. S9. CubA and CstR are required for TeoL to recruit OMVs from *P. aeruginosa*.
A. CubA and CstR are required for OMV (derives from *P. aeruginosa* strain) uptake in growth recovery. Relevant *C. necator* strains were grown until the stationary phase in NB medium and diluted to M9 containing EDDHA (5.5 μM) and OMVs (20 μg ml^{-1} of phospholipids). Cell growth was monitored by measuring the optical density at 600 nm (OD_{600}). **B.** TeoL bridges the interactions between OMVs and CubA or CstR. GST, GST-CubA, or GST-CstR were incubated with OMVs prepared from the OprF-VSVG expressing *P. aeruginosa* PAO1 in the presence or absence of His_6-TeoL. The formed protein–OMV complexes were captured by glutathione beads and detected by Western blotting. His_6-Hcp1 was used as a control. The vector corresponds to the pBBR1MCS-2 plasmid. Data are represented as mean values ± SD of three biological replicates, each with three technical replicates.

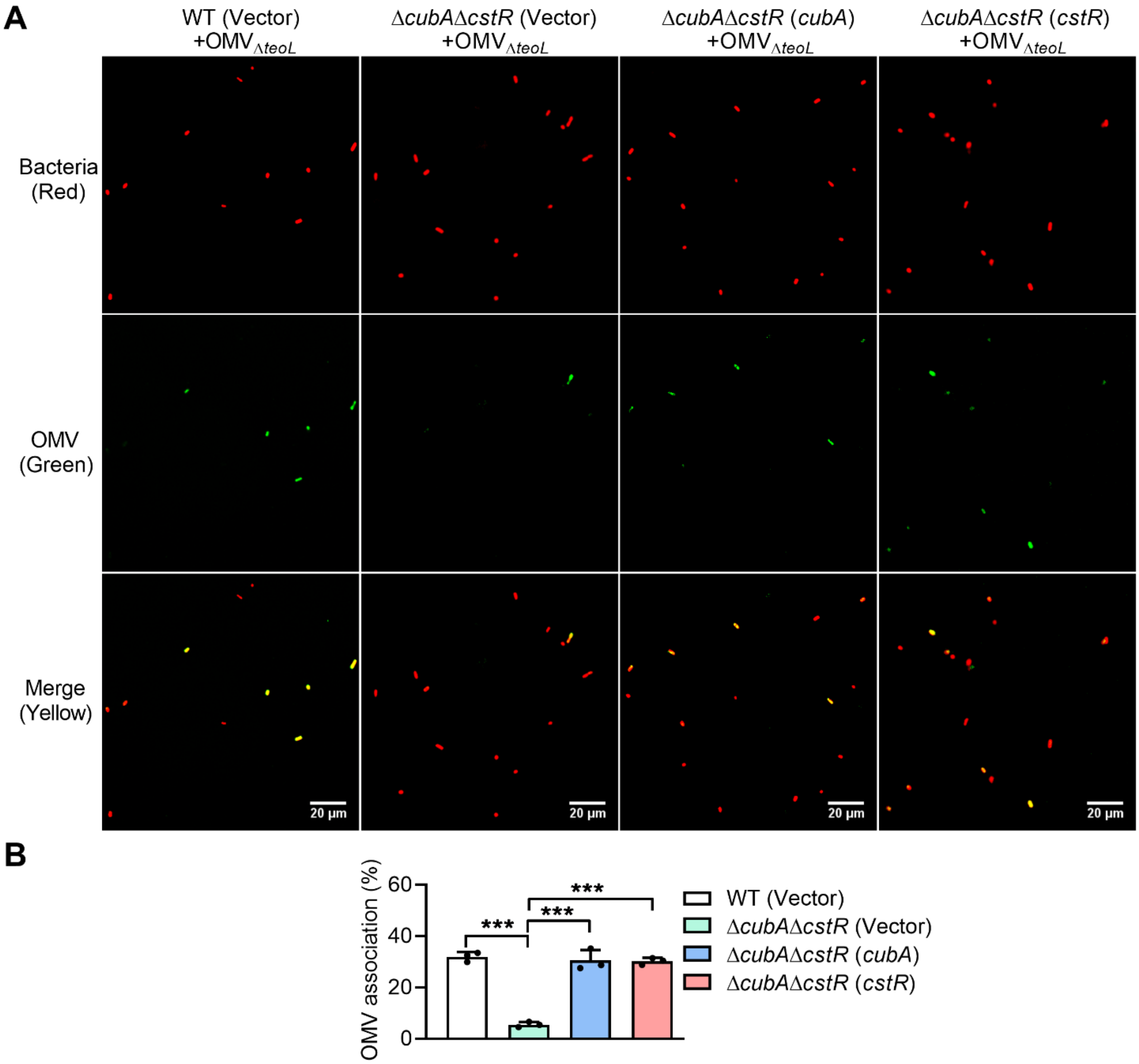

Fig. 10. CubA and CstR are required for TeoL-mediated OMV recruitment.
A-B. The mCherry-labeled relevant *C. necator* strains were washed three times with PBS and incubated with FITC-labeled OMVs (30 μg ml^{-1} of phospholipids) for 4 h at 30°C. After washed with PBS, the association between OMVs and indicated bacterial cells were observed by confocal microscopy (**A**) and the percentage of cells that exhibited both mCherry and FITC fluorescence were quantified (**B**). The pictures were taken and processed using ImageJ software. The vector corresponds to the pBBR1MCS-2 plasmid. Data are represented as mean values ± SD of three biological replicates, each with three technical replicates. ***, $p <$ 0.001.

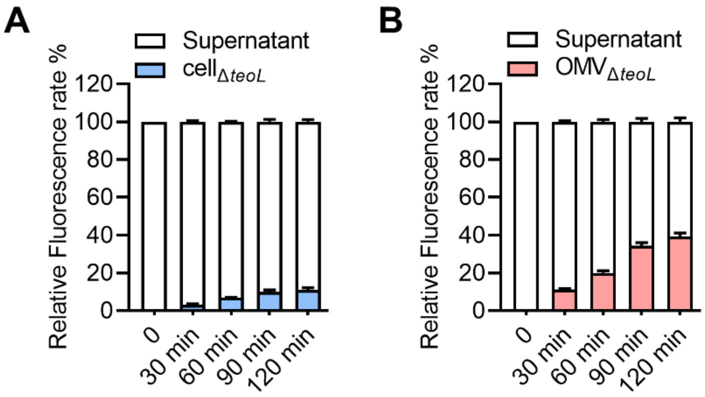

Fig. S11. TeoL proteins preferentially associated with OMVs.

A-B. GFP-TeoL protein was incubated with Δ*teoL* OMVs or Δ*teoL* cells containing equal amounts of LPS (30 μg ml^{-1} of phospholipid). After separated from the supernatant by ultracentrifugation (200,000 × *g*, 1 h, 4°C) at each time points, the Δ*teoL* OMVs or Δ*teoL* cells was resuspended in 1 ml PBS. The fluorescence of GFP-TeoL in supernatant, resuspended cell pellets and OMVs were determined using a fluorescence spectrometer, respectively, and the percentage of each component was calculated at each time point. Data are represented as mean values ± SD of three biological replicates, each with three technical replicates.

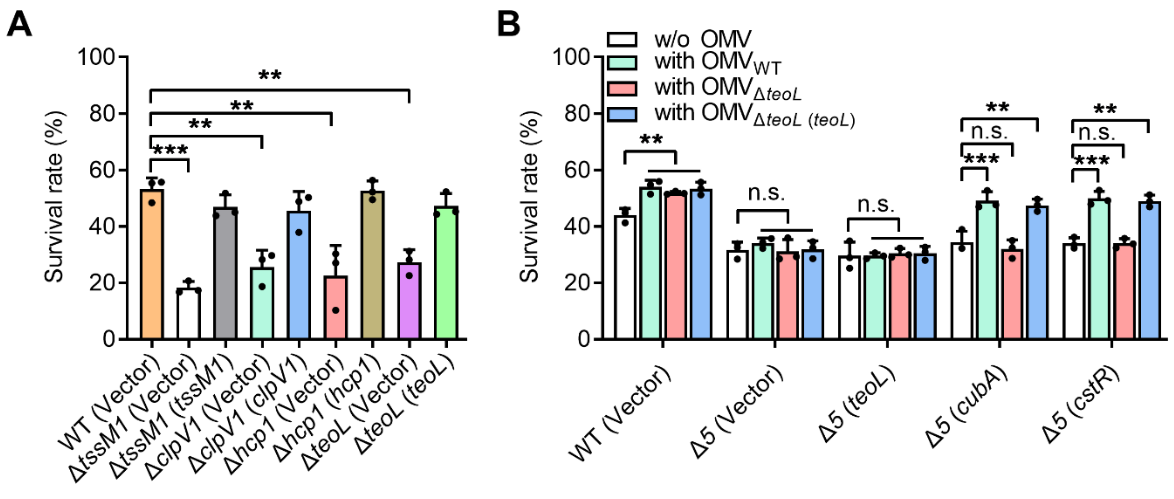

Fig. S12. The T6SS1 mediated OMVs recruitment system contributes to oxidative stress resistance.

A. T6SS1 participates in resistance to oxidative stress. The viability of mid-exponential-phase *C. necator* strains were determined after exposure to 0.1 mM H_2O_2 in M9 medium for 25 min.
B. TeoL recruits OMVs to resist oxidative stress. Indicated bacterial cells grown until stationary phase were exposed to 0.1 mM H_2O_2 in M9 medium for 25 min with or without OMVs (20 µg ml^{-1} of phospholipids) derived from *C. necator* WT, Δ*teoL* mutant and Δ*teoL*(*teoL*) complemented strains, respectively. Viability of the cells was determined by counting the colony forming units (CFUs). The vector corresponds to the pBBR1MCS-2 plasmid. Data are represented as mean values ± SD of three biological replicates, each of which was performed in three technical replicates. ***, $p < 0.001$; **, $p < 0.01$; n.s., not significant.

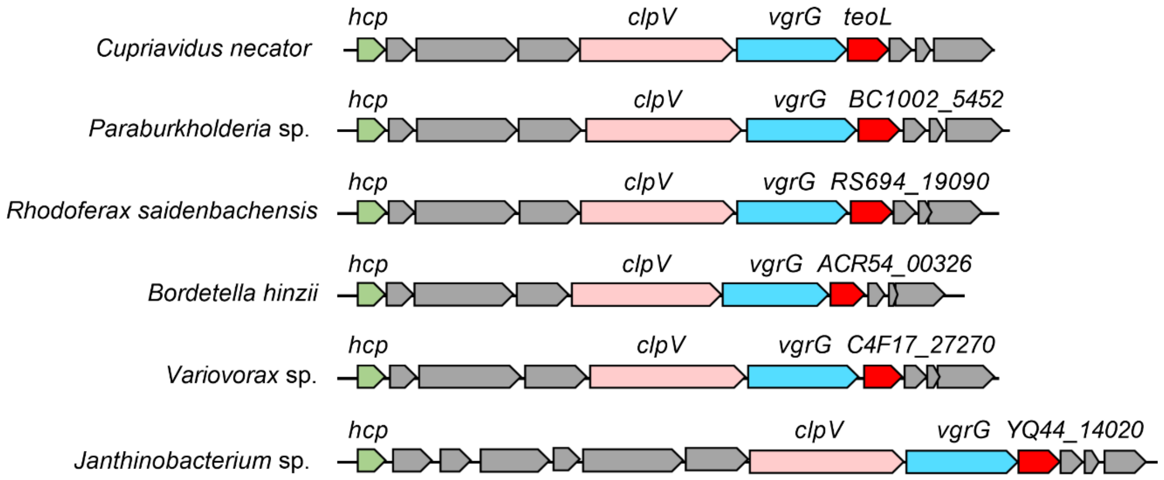

Fig. S13. Genomic arrangement of *teoL* ortholog genes from different species. Colors indicate *teoL* homologs (red), neighboring *vgrG* (blue), *clpV* (pink) and *hcp* (green) genes.

Supplementary Tables

Table S1. Bacterial strains and plasmids used in this study.

Strain or plasmid	Relevant characteristics	Reference
E. coli		
S17-1*λ pir*	*λ*-pir lysogen of S17-1, *thi pro hsdR hsdM*$^+$ *recA* RP4-Tc::Mu-Km::Tn7	[12]
BL21(DE3)	Host for expression vector pET28a	Novagen
XL-1 Blue	Host for expression vector pGEX-6p-1	Novagen
DH5α	FΦ80Δ*lacZ*ΔM15/Δ(*lacZYA-argF*)*U169recA1 endA1 hsdR17*	Lab stock
Y. pseudotuberculosis		
YPIII	Wild-type *Yersinia pseudotuberculosis* pIB1, Nal^r	[13]
YPIII(pBBR1MCS-5)	Wild-type YPIII containing pBBR1MCS-5, Nal^r, Gm^r	This study
YPIII(*ompW-vsvg*)	Wild-type YPIII containing pME6032-*ompW-vsvg*, Nal^r, Tc^r	This study
P. aeruginosa		
PAO1	Wild-type *Pseudomonas aeruginosa*	Lab stock
PAO1(*oprF-vsvg*)	Wild-type PAO1 containing pME6032-*oprF-vsvg*, Tc^r	This study
Δ*wzy*	*wzy* gene deleted in PAO1	This study
Δ*msbB*	*msbB* gene deleted in PAO1	This study
C. necator		
C. necator JMP134	Wild-type *Cupriavidus necator*	This study
Δ*fur*	*fur* gene deleted in *C. necator*	[1]
Δ*tssM1*	*tssM1* gene deleted in *C. necator*	This study
Δ*clpV1*	*clpV1* gene deleted in *C. necator*	This study
Δ*hcp1*	*hcp1* gene deleted in *C. necator*	This study
Δ*teoL*	*teoL* gene deleted in *C. necator*	This study
Δ*feoB*	*feoB* gene deleted in *C. necator*	This study
Δ*cubE*	*cubE* gene deleted in *C. necator*	[1]
Δ*cubA*	*cubA* gene deleted in *C. necator*	[1]
Δ*cstR*	*cstR* gene deleted in *C. necator*	This study
Δ*cubA*Δ*cstR* (Δ*2R*)	*cubA/cstR* genes deleted in *C. necator*	This study
Δ*cubE*Δ*feoB* (Δ*2Fe*)	*cubE/feoB* genes deleted in *C. necator*	This study
Δ*2Fe*Δ*clpV1*	*cubE/feoB/clpV1* genes deleted in *C. necator*	This study
Δ*2Fe*Δ*teoL*	*cubE/feoB/teoL* genes deleted in *C. necator*	This study
Δ*2Fe*Δ*2R*	*cubE/feoB/cubA/cstR* genes deleted in *C. necator*	This study
Δ*2Fe*Δ*2R*Δ*teoL* (Δ*5*)	*cubE/feoB/cubA/cstR/teoL* genes deleted in *C. necator*	This study
WT(P_{T6SS1}::*lacZ*)	Wild-type *C. necator* containing pK18-P_{T6SS1}::*lacZ*, Km^r	This study
WT(*mCherry*)	Wild-type *C. necator* containing pME6032-*mCherry*, Tc^r	This study
Δ*teoL*(*mCherry*)	Δ*teoL* containing pME6032-*mCherry*, Tc^r	This study
WT(Vector)	Wild-type *C. necator* containing pBBR1MCS-2/5, Km^r/Gm^r	[1]
Δ*fur*(Vector)	Δ*fur* containing pBBR1MCS-5, Gm^r	[1]
Δ*fur*(*fur*)	Δ*fur* containing pBBR1MCS-5-*fur*, Gm^r	[1]

Δ*tssM1*(Vector)	Δ*tssM1* containing pBBR1MCS-2, Km^r	This study
Δ*tssM1*(*tssM1*)	Δ*tssM1* containing pBBR1MCS-2-*tssM1*, Km^r	This study
Δ*clpV1*(Vector)	Δ*clpV1* containing pBBR1MCS-2, Km^r	This study
Δ*clpV1*(*clpV1*)	Δ*clpV1* containing pBBR1MCS-2-*clpV1*, Km^r	This study
Δ*hcp1*(Vector)	Δ*hcp1* containing pBBR1MCS-2, Km^r	This study
Δ*hcp1*(*hcp1*)	Δ*hcp1* containing pBBR1MCS-2-*hcp1*, Km^r	This study
Δ*teoL*(Vector)	Δ*teoL* containing pBBR1MCS-2, Km^r	This study
Δ*teoL*(*teoL*)	Δ*teoL* containing pBBR1MCS-2-*teoL*, Km^r	This study
WT(Vector, *teoL-vsvg*)	Wild-type *C. necator* containing pBBR1MCS-2 and pME6032-*teoL-vsvg*, Km^r, Tc^r	This study
Δ*tssM1*(Vector, *teoL-vsvg*)	Δ*tssM1* containing pBBR1MCS-2 and pME6032-*teoL-vsvg*, Km^r, Tc^r	This study
Δ*tssM1*(*tssM1, teoL-vsvg*)	Δ*tssM1* containing pBBR1MCS-2-*tssM1* and pME6032-*teoL-vsvg*, Km^r, Tc^r	This study
Δ*teoL*(*teoL-vsvg*)	Δ*teoL* containing pME6032-*teoL-vsvg*, Tc^r	This study
Δ*teoL*(*ompW-vsvg*)	Δ*teoL* containing pME6032-*ompW-vsvg*, Tc^r	This study
Δ*teoL*(*vgrG1-vsvg*)	Δ*teoL* containing pME6032-*vgrG1-vsvg*, Tc^r	This study
Δ*teoL*(*teoL-his*)	Δ*teoL* containing pME6032-*teoL-his*, Tc^r	This study
Δ*teoL*(*ompW-his*)	Δ*teoL* containing pME6032-*ompW-his*, Tc^r	This study
Δ*teoL*(*vgrG1-his*)	Δ*teoL* containing pME6032-*vgrG1-his*, Tc^r	This study
WT(*mCherry*)	Wild-type *C. necator* containing pME6032-*mCherry*, Tc^r	This study
Δ*teoL*(*mCherry*)	Δ*teoL* containing pME6032-*mCherry*, Tc^r	This study
Δ*2R*(Vector, *mCherry*)	Δ*2R* containing pBBR1MCS-2 and pME6032-*mCherry*, Km^r, Tc^r	This study
Δ*2R*(*cubA/cstR, mCherry*)	Δ*2R* containing pBBR1MCS-2-*cubA/cstR* and pME6032-*mCherry*, Km^r, Tc^r	This study
Δ*2Fe*(Vector)	Δ*2Fe* containing pBBR1MCS-2/5, Km^r/Gm^r	This study
Δ*2Fe*Δ*tssM1*(Vector)	Δ*2Fe*Δ*tssM1* containing pBBR1MCS-2/5, Km^r/Gm^r	This study
Δ*2Fe*Δ*tssM1*(*tssM1*)	Δ*2Fe*Δ*tssM1* containing pBBR1MCS-2-*tssM1*, Km^r	This study
Δ*2Fe*Δ*teoL*(Vector)	Δ*2Fe*Δ*teoL* containing pBBR1MCS-2/5, Km^r/Gm^r	This study
Δ*2Fe*Δ*teoL*(*teoL*)	Δ*2Fe*Δ*teoL* containing pBBR1MCS-2-*teoL*, Km^r	This study
Δ*2R*(Vector)	Δ*2R* containing pBBR1MCS-2, Km^r	This study
Δ*2R*(*cubA*)	Δ*2R* containing pBBR1MCS-2-*cubA*, Km^r	This study
Δ*2R*(*cstR*)	Δ*2R* containing pBBR1MCS-2-*cstR*, Km^r	This study
Δ*2Fe*Δ*2R*(Vector)	Δ*2Fe*Δ*2R* containing pBBR1MCS-2/5, Km^r/Gm^r	This study
Δ*2Fe*Δ*2R*(*cubA*)	Δ*2Fe*Δ*2R* containing pBBR1MCS-2-*cubA*, Km^r	This study
Δ*2Fe*Δ*2R*(*cstR*)	Δ*2Fe*Δ*2R* containing pBBR1MCS-2-*cstR*, Km^r	This study
Δ*5*(Vector)	Δ*5* containing pBBR1MCS-2, Km^r	This study
Δ*5*(*teoL*)	Δ*5* containing pBBR1MCS-2-*teoL*, Km^r	This study
Δ*5*(*cubA*)	Δ*5* containing pBBR1MCS-2-*cubA*, Km^r	This study
Δ*5*(*cstR*)	Δ*5* containing pBBR1MCS-2-*cstR*, Km^r	This study
Δ*2Fe*(pME6032)	Δ*2Fe* containing pME6032, Tc^r	This study
Δ*2Fe*Δ*teoL*(pME6032)	Δ*2Fe*Δ*teoL* containing pME6032, Tc^r	This study
Δ*2Fe*Δ*teoL*(pME6032-*teoL*)	Δ*2Fe*Δ*teoL* containing pME6032-*teoL*, Tc^r	This study
Δ*2Fe*Δ*2R*(pME6032)	Δ*2Fe*Δ*2R* containing pME6032, Tc^r	This study
Δ*2Fe*Δ*2R*(pME6032-*cubA*)	Δ*2Fe*Δ*2R* containing pME6032-*cubA*, Tc^r	This study
Δ*2Fe*Δ*2R*(pME6032-*cstR*)	Δ*2Fe*Δ*2R* containing pME6032-*cstR*, Tc^r	This study

**Plasmid**		
pK18*mobsacB*	*sacB*-based gene replacement vector, Km^r	[14]
pK18-Δ*tssM1*	Construct used for in-frame deletion of *tssM1*, Km^r	This study
pK18-Δ*clpV1*	Construct used for in-frame deletion of *clpV1*, Km^r	This study
pK18-Δ*hcp1*	Construct used for in-frame deletion of *hcp1*, Km^r	This study
pK18-Δ*teoL*	Construct used for in-frame deletion of *teoL*, Km^r	This study
pK18-Δ*cubE*	Construct used for in-frame deletion of *cubE*, Km^r	[1]
pK18-Δ*cubA*	Construct used for in-frame deletion of *cubA*, Km^r	[1]
pK18-Δ*feoB*	Construct used for in-frame deletion of *feoB*, Km^r	This study
pK18-Δ*cstR*	Construct used for in-frame deletion of *cstR*, Km^r	This study
pK18-P_{T6SS1P}::*lacZ*	For *T6SS1* promoter fusion to *C. necator*, Km^r	This study
pK18-Gm	pK18*mobsacB* carrying Gm coding region, Km^r, Gm^r	This study
pK18-Gm-Δ*wzy*	Construct used for in-frame deletion of *wzy*, Km^r, Gm^r	This study
pK18-Gm-Δ*msbB*	Construct used for in-frame deletion of *msbB*, Km^r, Gm^r	This study
pBBR1MCS-2	Broad-host-range vector, Km^r	[15]
pBBR1MCS-2-*tssM1*	*tssM1* cloned into pBBR1MCS-2 for complementation, Km^r	This study
pBBR1MCS-2-*clpV1*	*clpV1* cloned into pBBR1MCS-2 for complementation, Km^r	This study
pBBR1MCS-2-*hcp1*	*hcp1* cloned into pBBR1MCS-2 for complementation, Km^r	This study
pBBR1MCS-2-*teoL*	*teoL* cloned into pBBR1MCS-2 for complementation, Km^r	This study
pBBR1MCS-2-*cubA*	*cubA* cloned into pBBR1MCS-2 for complementation, Km^r	[1]
pBBR1MCS-2-*cstR*	*cstR* cloned into pBBR1MCS-2 for complementation, Km^r	This study
pBBR1MCS-5	Broad-host-range vector, Gm^r	[15]
pBBR1MCS-5-*fur*	*fur* cloned into pBBR1MCS-5 for complementation, Gm^r	[2]
pET28a	Expression vector with N-terminal His_6 affinity tag, Km^r	Novagen
pET28a-*fur*	pET28a carrying *fur* coding region, Km^r	[1]
pET28a-*hcp1*	pET28a carrying *hcp1* coding region, Km^r	This study
pET28a-*teoL*	pET28a carrying *teoL* coding region, Km^r	This study
pET28a-*GFP-teoL*	pET28a carrying *GFP-teoL* coding region, Km^r	This study
pGEX-6p-1	Expression vector with N-terminal GST tag, Amp^r	Novagen
pGEX-6p-1-*teoL*	pGEX-6p-1 carrying *teoL* coding region, Amp^r	This study
pGEX-6p-1-*cubA*	pGEX-6p-1 carrying *cubA* coding region, Amp^r	This study
pGEX-6p-1-*cstR*	pGEX-6p-1 carrying *cstR* coding region, Amp^r	This study
pME6032	Shuttle vector, Tc^r	[16]
pME6032-*mCherry*	pME6032 carrying *mCherry* coding region, Tc^r	This study
pME6032-*teoL*	pME6032 carrying *teoL* coding region, Tc^r	This study
pME6032-*cubA*	pME6032 carrying *cubA* coding region, Tc^r	This study
pME6032-*cstR*	pME6032 carrying *cstR* coding region, Tc^r	This study
pME6032-*teoL-vsvg*	pME6032 carrying *teoL-vsvg* coding region, Tc^r	This study
pME6032-*ompW-vsvg*	pME6032 carrying *ompW-vsvg* coding region, Tc^r	This study
pME6032-*vgrG1-vsvg*	pME6032 carrying *vgrG1-vsvg* coding region, Tc^r	This study
pME6032-*ypk_2049-vsvg*	pME6032 carrying *ypk_2049-vsvg* coding region, Tc^r	This study
pME6032-*PA1777-vsvg*	pME6032 carrying *PA1777-vsvg* coding region, Tc^r	This study

pME6032-*teoL-his*	pME6032 carrying *teoL-his* coding region, Tc^r	This study
pME6032-*ompW-his*	pME6032 carrying *ompW-his* coding region, Tc^r	This study
pME6032-*vgrG1-his*	pME6032 carrying *vgrG1-his* coding region, Tc^r	This study

*Nal^r, Gm^r, Km^r, Tc^r and Amp^r represent resistance to nalidixic acid, gentamicin, kanamycin, tetracycline and ampicillin, respectively.

Table S2. Primers used in this study.

Primers	5'-3' sequence	Function
tssM1-1F-BglII	GGAAGATCTGACCCGAAGCCGAGGACGACGAT	To generate
tssM1-1R	GCACGCCGATCGAGATCAGGAAGAG	pK18-Δ*tssM1*
tssM1-2F	**CTCTTCCTGATCTCGATCGGCGTGC**TCCCGACCCGTGCTCCCGACT	
tssM1-2R-SalI	ACGCGTCGACGCATCCATGACCGCCGACGC	
clpV1-1F-BglII	GGAAGATCTCCTGTCCTTCCTCTATCGCGGTCGTG	To generate
clpV1-1R	CGGATTAAGCCGGCTCAGCAGTGTG	pK18-Δ*clpV1*
clpV1-2F	**CACACTGCTGAGCCGGCTTAATCCG**CCTGCGGAGGCCTGAGGCAATGA	
clpV1-2R-SalI	ACGCGTCGACGGCATCCGAGGCATCCTTGTGGC	
hcp1-1F-BglII	GGAAGATCTACGGCACCGATCACGGCAAGTACC	To generate
hcp1-1R	ATCTGGATCTTGTCTTCATGGCCGGTG	pK18-Δ*hcp1*
hcp1-2F	**CACCGGCCATGAAGACAAGATCCAGAT**CCGCTTTCGGCTGGGACCTGG	
hcp1-2R-SalI	ACGCGTCGACGCGCCGGGCAGACATTCTCCAG	
teoL-1F-BglII	GGAAGATCTCAAGTTCCACTGGGACCAGGCGC	To generate
teoL-1R	CTCGCGTTGGCGGCAATGTCC	pK18-Δ*teoL*
teoL-2F	**GGACATTGCCGCCAACGCGAG**CAACGCCTCGAGCGCTGGGTC	
teoL-2R-SalI	ACGCGTCGACTGGCTCAGGCGCGAAGAGAATGC	
feoB-1F-EcoRI	CCGGAATTCCGAACTGGGCAAGACCAACGAGC	To generate
feoB-1R	AGGGTCAATGCGGTCATGGGG	pK18-Δ*feoB*
feoB-2F	**CCCCATGACCGCATTGACCCT**GCAGGGAGGATGTCACTGATGGC	
feoB-2R-BamHI	CGCGGATCCCGCCTGCTGCTGGAGGTCACC	
cstR-1F-BglII	GGAAGATCTCCCTATGCTTCCCTCTACCG	To generate
cstR-1R	TCGTCCTTCGTGCTCGTCAC	pK18-Δ*cstR*
cstR-2F	**GTGACGAGCACGAAGGACGA**CCAGAACCTGTTCGACAAGA	
cstR-2R-EcoRI	CCGGAATTCAGCCATGATGCTGCAGATTC	
lacZ-F-XbaI	TGCTCTAGAATGACCATGATTACGGATTCAC	To generate
lacZ-R-SphI	GTGCGCATGCTTAAGCGACTTCATTCACCTGA	pK18-P_{T6SS1}::*lacZ*
P_{T6SS1}-F-BamHI	CGCGGATCCTTGTTGGTAATTGTTGGATTCA	
P_{T6SS1}-R-XbaI	TGCTCTAGACAGGCTATTGGACATGGAACTC	
Gm-F-BglII	AGGATCTGATGGCGCAGGGGATCAAGATCTGTCGATGTTTGATGTTATGGAGCA	To generate pK18-Gm
Gm-R-BglII	CGATCCTCATCCTGTCTCTTGATCAGATCTGGTGGCGGTACTTGGGTCG	
wzy-1F-EcoRI	GGAAACAGCTATGACCATGATTACGAATTCCAGACTTCGATGCAATCAACG	To generate
wzy-1R	CCCACACTGTCGCTGAAAAGAAAG	pK18-Gm-Δ*wzy*
wzy-2F	**CTTTCTTTTCAGCGACAGTGTGGG**TCCTTGCGCTAATTTCTTTTCCACTC	
wzy-2R-BamHI	GCATGCCTGCAGGTCGACTCTAGAGGATCCCCAGATCCGCAGGCAG	

	AGAAAAC	
msbB-1F-EcoRI	CCGGAATTCGACCGCGGCCAGCACGATC	To generate
msbB-1R	CCATGGACGGTTCCGACGACG	pK18-Gm-Δ*msbB*
msbB-2F	**CGTCGTCGGAACCGTCCATGG**ATCCGAAACGGCGCAAGCG	
msbB-2R-BamHI	CGCGGATCCGCCGAAGTATTCGCGGTTGATCC	
tssM1-F-HindIII	CCCAAGCTTATGCTGACCAGTAATCTCTTCCTGATCTCGA	To generate
tssM1-R-XbaI	TGCTCTAGATCACAGCGGGTTCCAGTCGGGAG	pBBR1MCS-2-*tssM1*
clpV1-F-BamHI	CGCGGATCCATGGACATCGATATCCGCACACTGCTG	To generate
clpV1-R-XbaI	TGCTCTAGATCAGGCCTCCGCAGGCGGC	pBBR1MCS-2-*clpV1*
hcp1-F-HindIII	CCCAAGCTTATGGACACCATCATCCTCGAGATCACTGAT	To generate
hcp1-R-XbaI	TGCTCTAGATCAGGAAACGGCCTTGTTGGTAGCC	pBBR1MCS-2-*hcp1*
teoL-F-EcoRI	CCGGAATTCATGCACGCCACCGCCCCCCTG	To generate
teoL-R-BamHI	CGCGGATCCTCAGCCCCTGACCCAGCGCTCGA	pBBR1MCS-2-*teoL*
cstR-F-HindIII	CCCAAGCTTTTGAACCCGCTCGCCGCAGC	To generate
cstR-R-XbaI	CTAGTCTAGATCAGAACCTGAAGTTCGCCG	pBBR1MCS-2-*cstR*
teoL-F-BamHI	CGCGGATCCATGCACGCCACCGCCCCCCTGGAC	To generate
teoL-R-SalI	ACGCGTCGACTCAGCCCCTGACCCAGCGCTCGAGG	pET28a-*teoL*
GFP-F-BamHI	CGCGGATCCATGGTGAGCAAGGGCGAGGAG	pGEX-6p-1-*teoL*
GFP-R-BamHI	CGCGGATCCCTTGTACAGCTCGTCCATGCCGA	pET28a-*GFP*-*teoL*
hcp1-F-BamHI	CGCGGATCCATGGACACCATCATCCTCG	To generate
hcp1-R-SalI	ACGCGTCGACTCAGGAAACGGCCTTGTTGG	pET28a-*hcp1*
cubA-F-BglII	GGAAGATCTATGCCGGTCCCCGGTCAGAC	To generate
cubA-R-SalI	CCGCTCGAGTCAGAATTCAACGGTGGCGG	pGEX-6p-1-*cubA*
cstR-F-BamHI	CGCGGATCCTTGAACCCGCTCGCCGCAGC	To generate
cstR-R-EcoRI	CCGGAATTCTCAGAACCTGAAGTTCGCCG	pGEX-6p-1-*cstR*
mCherry-F-EcoRI	CCGGAATTCATGGTGAGCAAGGGCGAGGAG	To generate
mCherry-R-BamHI	CGCGGATCCCTTGTACAGCTCGTCCATGCCG	pME6032-*mCherry*
teoL-F-EcoRI	CCGGAATTCATGCACGCCACCGCCCCCCTG	To generate
teoL-R-*vsvg*-BglII	GGAAGATCTTCATTTTCCTAATCTATTCATTTCAATATCTGTATAGCCC	pME6032-*teoL*-*vsvg*
	CTGACCCAGCGCTCGAGGC	pME6032-*teoL*-*his*
teoL-R-*his*-BamHI	CGCGGATCCTCAGTGGTGGTGGTGGTGGTGAGATCTGCCCCTGACC	
	CAGCGCTCGAGGC	
ompW-F-EcoRI	CCGGAATTCATGAAATCGAACTATAAGAAGATGCTGGCG	To generate
ompW-R-BglII	GAAGATCTGAACTTGTAGCCCACGCTCAGGAAAGT	pME6032-*ompW*-*vsvg*
		pME6032-*ompW*-*his*
vgrG1-F-EcoRI	CCGGAATTCATGATTCCGGCCACCGCCAC	To generate
vgrG1-R-BglII	GAAGATCTGTTCAGTTTCACCATGGCGCCCT	pME6032-*vgrG1*-*vsvg*
		pME6032-*vgrG1*-*his*
ypk_2049-F-EcoRI	CCGGAATTCATGAAAAAAATCACTTTGGCACTGCTCG	To generate
ypk_2049-R-BglII	GGAAGATCTAAAACGATAACCCGCGCCAAACA	pME6032-*ompW*-*vsvg*
PA1777-F-EcoRI	CCGGAATTCATGAAACTGAAGAACACCTTAGGCGTTGT	To generate
PA1777-R-BglII	GGAAGATCTATGAAACTGAAGAACACCTTAGGCGTTGT	pME6032-*oprF*-*vsvg*
cubA-F-SacI	CGAGCTCATGCCGGTCCCCGGTCAGAC	To generate

cubA-R-BglII	GAAGATCTTCAGAATTCAACGGTGGCGG	pME6032-*cubA*
cstR-F-EcoRI	CCGGAATTCTTGAACCCGCTCGCCGCAGC	To generate
cstR-R-BamHI	CGCGGATCCTCAGAACCTGAAGTTCGCCG	pME6032-*cstR*
T6SS1-EMSA-F	AAATGCATCGCTCTCAGA	EMSA
T6SS1-EMSA-R	AATCTTTGTGTTTCTGTTG	
Control-F	AGCTTGCGGCCGCGTCGA	
Control-R	AGTTGGAGCCGGTCTGCT	
Q16S-F	GGGGAGTACGGTCGCAAGA	qRT-PCR
Q16S-R	ATGTCAAGGGTAGGTAAGGTTT	
QtssM1-F	AGGGCATCTCGTGGCACTTCT	
QtssM1-R	AACTCGTCCCAGGGCTTTTCAT	
QvgrG1-F	TGACGAGACCCGCACCAA	
QvgrG1-R	TGACCAGATCGCCAGACACC	
QclpV1-F	GTGCTGCTCGACGAAATGGA	
QclpV1-R	GGATGACCGTGTTGCGGAAGT	
Qhcp1-F	CCGCTGATGACCTACACGC	
Qhcp1-R	CTCGGTGAAATCCAGACAGAACG	
QteoL-F	CTGACGCTGTCCGAGCCCTTTC	
QteoL-R	GCGGCTTGCCATCGGTGTAG	
KanR-F	GTTGTCACTGAAGCGGGAAG	
KanR-R	ATCCTGATCGACAAGACCGG	

Underlined sites Indicate restriction enzyme cutting sites added for cloning. Letters in boldface denote the annealing regions for overlap PCR.

Supplementary References

1. Li C, Zhu L, Pan D, Li S, Xiao H, Zhang Z, et al. Siderophore-mediated iron acquisition enhances resistance to oxidative and aromatic compound stress in *Cupriavidus necator* JMP134. Appl Environ Microbiol. 2019;85(1).
2. Lin J, Zhang W, Cheng J, Yang X, Zhu K, Wang Y, et al. A *Pseudomonas* T6SS effector recruits PQS-containing outer membrane vesicles for iron acquisition. Nat Commun. 2017;8:14888.
3. Miller JH. A short course in bacterial genetics: a laboratory manual and handbook for *Escherichia coli* and related bacteria. Cold Spring Harbor, NY, USA: Cold Spring Harbor Laboratory Press. 1992.
4. Shen X, Banga S, Liu Y, Xu L, Gao P, Shamovsky I, et al. Targeting eEF1A by a *Legionella pneumophila* effector leads to inhibition of protein synthesis and induction of host stress response. Cell Microbiol. 2009;11(6):911-26.
5. Xu S, Peng Z, Cui B, Wang T, Song Y, Zhang L, et al. FliS modulates FlgM activity by acting as a non-canonical chaperone to control late flagellar gene expression, motility and biofilm formation in *Yersinia pseudotuberculosis*. Environ Microbiol. 2014;16(4):1090-104.
6. Xu L, Shen X, Bryan A, Banga S, Swanson MS, Luo ZQ. Inhibition of host vacuolar H^+-ATPase activity by a *Legionella pneumophila* effector. PLoS Pathog. 2010;6(3):e1000822.
7. Wang T, Si M, Song Y, Zhu W, Gao F, Wang Y, et al. Type VI secretion system transports Zn^{2+} to combat multiple stresses and host immunity. PLoS Pathog. 2015;11(7):e1005020.
8. DebRoy C, Roberts E, Davis M, Bumbaugh A. Multiplex polymerase chain reaction assay for detection of nonserotypable Shiga toxin-producing *Escherichia coli* strains of serogroup O147. Foodborne Pathog Dis. 2010;7(11):1407-14.
9. Schilling O, Vogel A, Kostelecky B, Natal da Luz H, Spemann D, Spath B, et al. Zinc- and iron-dependent cytosolic metallo-beta-lactamase domain proteins exhibit similar zinc-binding affinities, independent of an atypical glutamate at the metal-binding site. Biochem J. 2005;385(Pt 1):145-53.
10. Si M, Zhao C, Burkinshaw B, Zhang B, Wei D, Wang Y, et al. Manganese scavenging and oxidative stress response mediated by type VI secretion system in *Burkholderia thailandensis*. Proc Natl Acad Sci U S A. 2017;114(11):E2233-E42.
11. Hellman LM, Fried MG. Electrophoretic mobility shift assay (EMSA) for detecting protein-nucleic acid interactions. Nat Protoc. 2007;2(8):1849-61.
12. Simon R, Priefer U, Pühler A. A broad host range mobilization system for *in vivo* genetic engineering: transposon mutagenesis in Gram negative bacteria. Bio/Technology. 1983;1(9):784.
13. Rosqvist R, Skurnik M, Wolf-Watz H. Increased virulence of *Yersinia pseudotuberculosis* by two independent mutations. Nature. 1988;334(6182):522-4.
14. Schafer A, Tauch A, Jager W, Kalinowski J, Thierbach G, Puhler A. Small mobilizable multi-purpose cloning vectors derived from the *Escherichia coli* plasmids pK18 and pK19: selection of defined deletions in the chromosome of *Corynebacterium glutamicum*. Gene. 1994;145(1):69-73.
15. Kovach ME, Elzer PH, Hill DS, Robertson GT, Farris MA, Roop RM, 2nd, et al. Four new derivatives of the broad-host-range cloning vector pBBR1MCS, carrying different antibiotic-resistance cassettes. Gene. 1995;166(1):175-6.
16. Heeb S, Blumer C, Haas D. Regulatory RNA as mediator in GacA/RsmA-dependent global control of exoproduct formation in *Pseudomonas fluorescens* CHA0. J Bacteriol. 2002;184(4):1046-56.
